# Long-Term Overconsumption of Fat and Sugar Causes a Partially Reversible Pre-inflammatory Bowel Disease State

**DOI:** 10.3389/fnut.2021.758518

**Published:** 2021-11-18

**Authors:** Djésia Arnone, Marie Vallier, Sébastien Hergalant, Caroline Chabot, Ndeye Coumba Ndiaye, David Moulin, Anda-Maria Aignatoaei, Jean-Marc Alberto, Huguette Louis, Olivier Boulard, Camille Mayeur, Natacha Dreumont, Kenneth Peuker, Anne Strigli, Sebastian Zeissig, Franck Hansmannel, Matthias Chamaillard, Tunay Kökten, Laurent Peyrin-Biroulet

**Affiliations:** ^1^Inserm U1256, Nutrition Genetics and Exposition NGERE, Université de Lorraine, Nancy, France; ^2^Section of Evolutionary Medicine, Institute for Experimental Medicine, Kiel University and Max Planck Institute for Evolutionary Biology, Plön, Germany; ^3^CHRU-Nancy, Pediatric Hepato-Gastroenterology and Nutrition Unit, Department of Child Medicine and Clinical Genetics, Inserm U1256, Université de Lorraine, Nancy, France; ^4^IMoPA, UMR7365 CNRS-Université de Lorraine, CHRU de Nancy, Contrat d'interface, Nancy, France; ^5^Department of Anatomopathology, CHRU de Nancy, Nancy, France; ^6^Department Inserm UMRS_1116 DCAC, Université de Lorraine, Nancy, France; ^7^Cytometry Core Facility, UMS2008 IBSLor (CNRS-Université de Lorraine-INSERM), Campus Brabois-Santé, Nancy, France; ^8^Laboratory of Cell Physiology, INSERM U1003, University of Lille, Lille, France; ^9^Micalis Institute, INRAE, AgroParisTech, Université Paris-Saclay, Jouy-en-Josas, France; ^10^Center for Regenerative Therapies, Technische Universität (TU) Dresden, Dresden, Germany; ^11^Department of Medicine I, University Medical Center Dresden, Technische Universität (TU) Dresden, Dresden, Germany; ^12^Department of Gastroenterology, CHRU-Nancy, Université de Lorraine, Nancy, France

**Keywords:** diet-induced, IBD-inflammatory bowel diseases, colitis, gut homeostasis, high-fat high-sucrose diet

## Abstract

Nutrition appears to be an important environmental factor involved in the onset of inflammatory bowel diseases (IBD) through yet poorly understood biological mechanisms. Most studies focused on fat content in high caloric diets, while refined sugars represent up to 40% of caloric intake within industrialized countries and contribute to the growing epidemics of inflammatory diseases. Herein we aim to better understand the impact of a high-fat-high-sucrose diet on intestinal homeostasis in healthy conditions and the subsequent colitis risk. We investigated the early events and the potential reversibility of high caloric diet-induced damage in mice before experimental colitis. C57BL/6 mice were fed with a high-fat or high-fat high-sucrose or control diet before experimental colitis. In healthy mice, a high-fat high-sucrose diet induces a pre-IBD state characterized by gut microbiota dysbiosis with a total depletion of bacteria belonging to Barnesiella that is associated with subclinical endoscopic lesions. An overall down-regulation of the colonic transcriptome converged with broadly decreased immune cell populations in the mesenteric lymph nodes leading to the inability to respond to tissue injury. Such *in-vivo* effects on microbiome and transcriptome were partially restored when returning to normal chow. Long-term consumption of diet enriched in sucrose and fat predisposes mice to colitis. This enhanced risk is preceded by gut microbiota dysbiosis and transcriptional reprogramming of colonic genes related to IBD. Importantly, diet-induced transcriptome and microbiome disturbances are partially reversible after switching back to normal chow with persistent sequelae that may contribute to IBD predisposition in the general population.

**Graphical Abstract:**
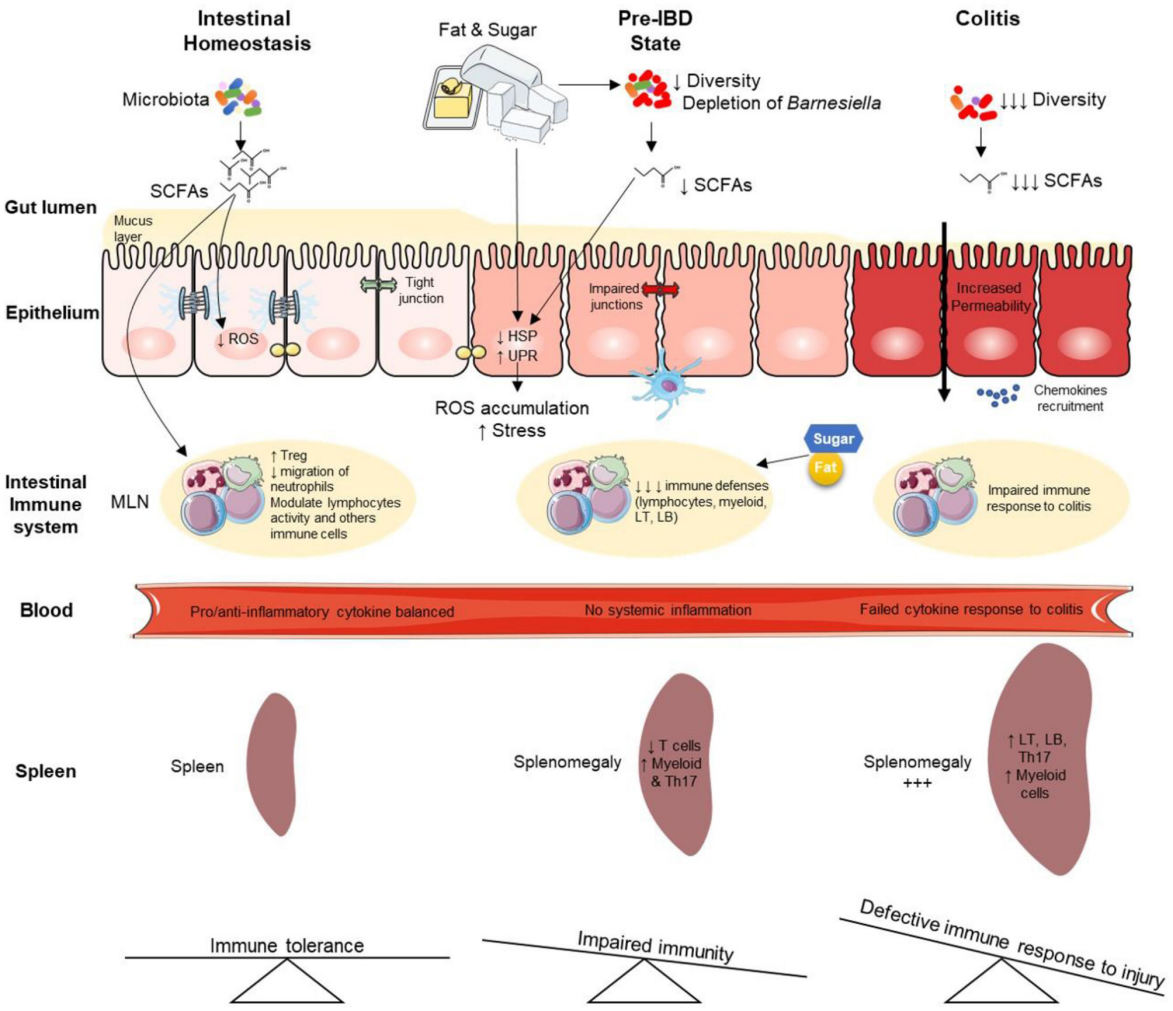
Overview of high-fat high-sucrose-mediated predisposition to colitis and aggravating effects during established colitis. IBD, Inflammatory Bowel Disease; LT, lymphocyte T; LB, lymphocyte B; HSP, heat shock proteins; MLN, mesenteric lymph node; ROS, reactive oxygen species; UPR, unfolded protein response; SCFAs, Short Chain Fatty Acids.

## Introduction

Inflammatory Bowel Diseases (IBD) are chronic inflammatory diseases of the gastrointestinal tract with increasing incidence worldwide (1). Dietary patterns in industrialized countries are characterized by overconsumption of fats and sugars in the total caloric availability (2), the so-called Western Diet (WD). This consumption has increased by about 10-fold over the last century in industrialized countries (3–5). Many epidemiological studies concluded that WD is associated with an increased risk of developing several human diseases, including inflammatory conditions such as IBD (2, 6). Recent international recommendations expressed concerns about sugar consumption in Westernized societies as they represent quantities with no precedent during hominin evolution (3, 4). In both adults and children, the World Health Organization (WHO) strongly recommends reducing the intake of free sugars to less than 10% of total energy intake and suggests a further reduction to below 5% (3–5).

Animal studies have shown that WD is responsible for deleterious effects on intestinal permeability, gut microbiota, risk of infection, and experimental colitis (7–9). While the consequences of high-fat (HF) diets on gut microbiota dysbiosis are well studied, few studies focused on sugar's specific role on intestinal homeostasis (10). A landmark study showed that experimental type 2 diabetes drives intestinal barrier dysfunction by transcriptional reprogramming of intestinal epithelial cells and alteration of tight and adherens junctions integrity (11). A high sucrose diet (enriched with 55% sucrose) was shown to enhance the susceptibility to experimental colitis, but experiments were performed over a very short period (only a few days) and at a very high dose (12). More recently, investigated sugar-induced exacerbation of colitis when adding 10% glucose or fructose in drinking water (mimicking sugar-sweetened beverages), dietary sugars quickly altered gut microbial ecology (13). In agreement with these findings, rectal insulin instillation inhibited inflammation in chemically-induced colitis in mice (14). Although growing evidence underpins the pro-inflammatory effect of sugars (15, 16), the long-term impact of combined excessive intake of dietary sugar and fat due to ultra-processed food overconsumption (enriched in added sugars) on healthy intestine and the underlying molecular mechanisms remain poorly described (9). Also, the potential reversibility of high caloric diet-induced damage is unknown. Here we investigated the effects of long-term exposure to both dietary sugar and fat at a moderate dose (30% sucrose, 25% fat) on the healthy intestine. We showed that combined sugar and fat in diet induces a pre-IBD state characterized by decreased immune cell population in the mesenteric lymph nodes (MLNs) and associated with a gut microbiota dysbiosis, with total depletion of bacteria belonging to *Barnesiella* [a genus hinted to be protective against colitis (17)] associated with subclinical endoscopic lesions.

Furthermore, disease risk was heightened in Germ-Free (GF)-mice that were colonized with microbiota from high-fat high-sucrose (HFHS)-fed mice, demonstrating that gut microbiota dysbiosis played a critical role in the diet-induced colitis predisposition. This pre-IBD state is also associated with an overall down-regulation of the colonic transcriptome characterized by differential expression of stress and autophagy-related genes. Both transcriptome and microbiome alterations induced by HFHS were partially reversible, with persistent sequelae when mice were refeeding with the normal chow diet.

Our results are in line with the beneficial effect of reducing dietary sugars (10, 18–22). These findings support WHO recommendations about the urgent need to reduce sugar consumption in the general population, e.g., by limiting processed food consumption (23).

## Results

### High-Fat High-Sucrose Diet Causes a Pre-inflammatory Bowel Disease State

**Figure 1:**
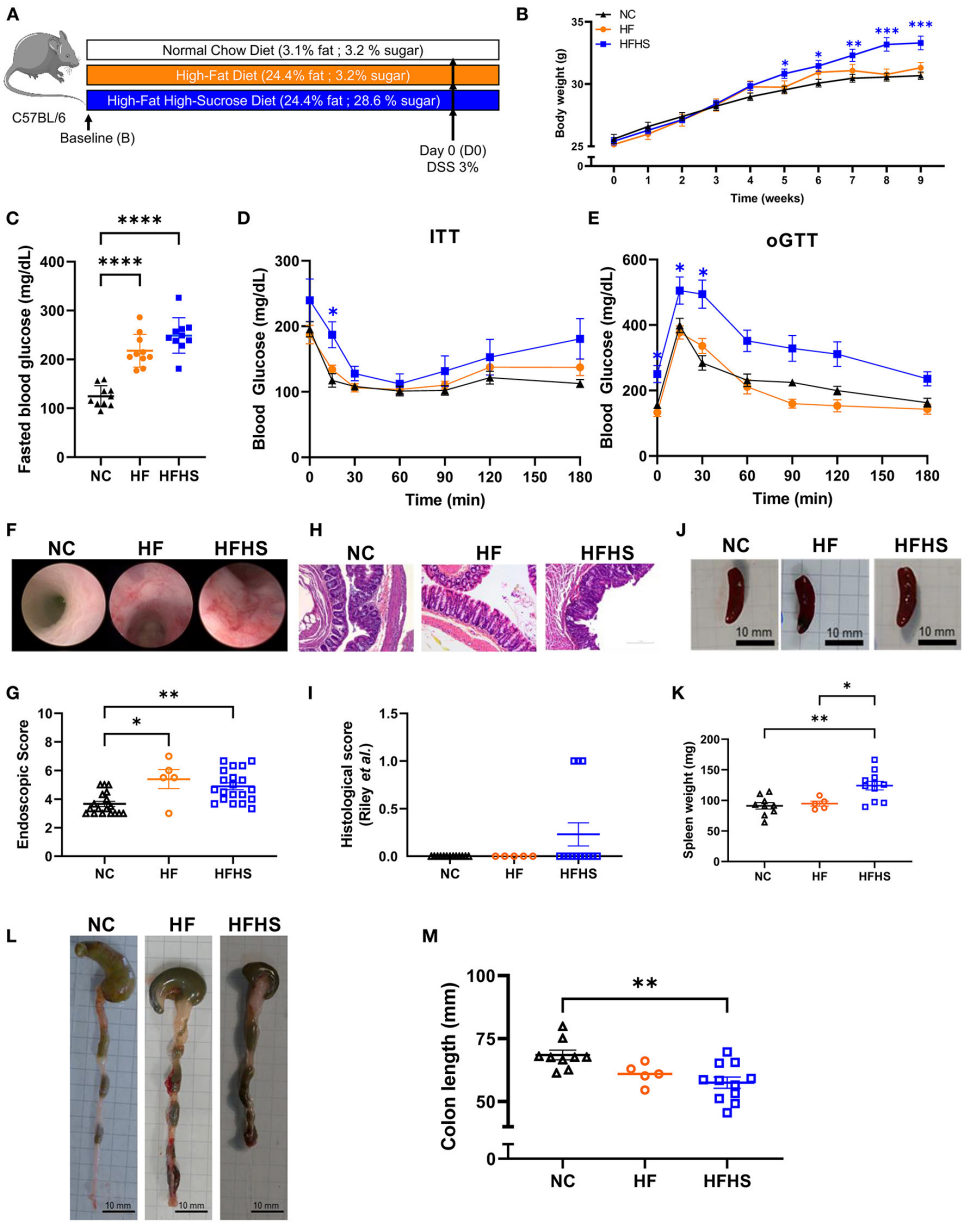
Additive effects of sucrose and fat in healthy mice. **(A)** Experimental design. Mice were fed 8 weeks with normal chow diet (NC) or High-Fat (HF) diet or High-Fat High-Sucrose (HFHS). **(B)** Body weight of HSHF-fed mice (*N* = 40) was higher than HF-fed mice (*N* = 10) or NC-fed mice (*N* = 36). **(C)** Fasting blood glucose levels in HFHS, HF and NC-fed mice (*N* = 10 /group). **(D)** Insulin tolerance test (ITT) after 8 weeks of diet (0.75 U/kg insulin, ip). **(E)** Oral glucose tolerance test (oGGT) after 8 weeks of diet (2 g/kg per os) for HFHS, HF and NC-fed mice (*N* = 5/group). **(F)** Representative colonoscopy images **(G)** Ulcerative colitis endoscopic index of severity (UCEIS) **(H)** Representative sections of colon, arrows show epithelial dystrophies. Scale bars, 0.10 mm. **(I)** Histological scores. **(J)** Gross pictures of spleens, scale bars: 10 mm. **(K)** Spleen weight to body weight ratio. **(L)** Gross pictures of colons. Scale bars, 10 mm. **(M)** Colon lengths. Data are given as means +/- SEM. *p < 0.05, **p < 0.01, ***p < 0.001, ****p < 0.0001 by ANOVA (two-way for B, D and E) for parametric data or Kruskal-Wallis if not.

Mice were fed with a Normal Chow diet (NC), High Fat (HF, 25% fat) or High-Fat High-Sucrose diet (HFHS, 25% fat, 30% sucrose) for 8 weeks (Figure 1A). No differences were observed in food and water consumption, but weight gain were increased in HFHS-fed mice and blood glucose were increased in both HFHS and HF-fed compared to NC-fed mice (Figures 1B–D). Dietary intake was quite stable (2–4 g/day) under ad libitum conditions, whereas energy intake was higher after HFHS (4,775.7 kcal/kg) and HF (4,649 kcal/kg) than NC (3,339 kcal/kg), as expected. Consequently, body weight over the 8-week dietary intervention was significantly higher in HFHS-fed mice than NC-fed mice or the HF (Figure 1B). Blood glucose levels were measured after eight weeks of diets and 12-h fasting (Figure 1C), and metabolic tests (oGTT and ITT) confirmed that only HFHS diet induced prediabetes characterized by reduced impaired glucose tolerance by insulin resistance (Figures 1D,E).

Unexpectedly, endoscopic lesions were observed in the colonic mucosa of HFHS-fed and HF-fed mice not treated with Dextran-sulfate sodium (DSS) (Figure 1F), and the endoscopic score (Figure 1G, Supplementary Table 1) was significantly higher (1.4-fold increase; *p* = 0.0089) than in NC-fed mice not treated with DSS. These endoscopic lesions were associated with a thinning and shortening of the colon length (-10.9 mm; *p* = 0.012) (Figures 1L,M). At the histological level (Figures 1H,I, Supplementary Table 2), some HFHS-fed animals showed a chronic inflammatory cell infiltrate (lymphoplasmacytic, Supplementary Table 3), with no significant increase in the global score (Figure 1I).

Then we used DSS colitis as a control and we confirmed that clinical manifestations of colitis appeared earlier in HFHS-fed mice (day 3) and that the colitis was more severe, as reflected by decreased body weight (Supplementary Figure 1A) and the higher Disease Activity Index (DAI) in the HFHS DSS^+^ group compared with NC DSS^+^ animals (3.8 vs. 2.7 at day 10, respectively; *p* < 0.0001) (Supplementary Figure 1B). Likewise, the endoscopic score indicated a severe colonic inflammation (Supplementary Figures C,D) that was also confirmed histologically (Supplementary Figures 1E,F, Supplementary Table 1). This was further supported by the thinning and shortening of the colon length (Supplementary Figures 1G,H). In agreement with these findings, an enlarged spleen was observed in either HFHS DSS^+^ mice (about larger 3-fold maximum size compared to NC DSS^+^; *p* = 0.0001) (Supplementary Figures 1K,L) or HFHS DSS^−^ mice (1.5-fold on average compared to NC DSS^−^; *p* = 0.11; Figures 1J,K). In contrast, no significant decrease of colon length was observed in HF-fed mice, even with colitis (Supplementary Figures 1I,J). On day 5 of DSS-treatment, intestinal permeability was measured by FITC-Dextran, showing that only HFHS-fed mice treated with DSS had increased permeability compared to both NC or HF DSS^+^ group (Supplementary Figure 1D). Furthermore, HFHS increased early mortality during DSS-colitis whereas HF-fed mice have similar mortality to NC-fed (30% of HFHS-fed mice from the 3rd day; *p* = 0.0066 vs. 20% of NC or HF-fed mice died after 7 days; Supplementary Figure 1C).

Altogether, these data showed that sucrose added to fat (rather than fat alone) aggravates the severity of DSS-induced colitis clinically, endoscopically, and histologically. Under physiological conditions, such diet induces a pre-IBD state characterized by endoscopic lesions, reduced colon length, and inflammatory cell infiltration in the colonic mucosa.

### Specific Effects of Sucrose on the Intestinal and Systemic Immune Homeostasis of Healthy Mice

**Figure 2:**
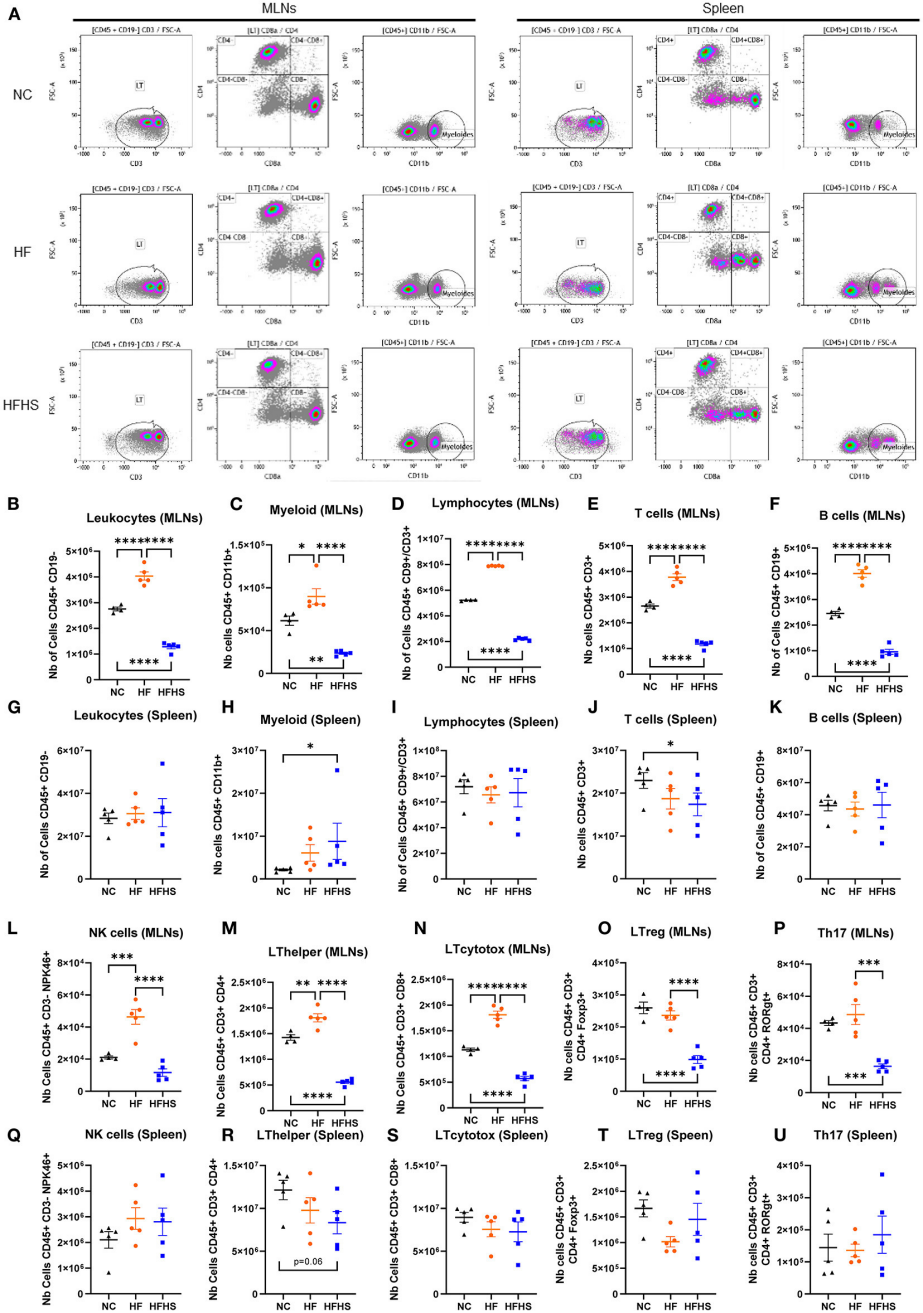
Sucrose repressed immune cell levels in mesenteric lymph nodes and spleen. **(A)** Representative flow cytometry recording mesenteric lymph nodes (MLNs) and spleen. **(B–U)** Relative abundance of indicated immune cell populations MLNs or spleen for mice fed with normal chow diet (NC, *N* = 5), high-fat diet (HF, *N* = 5), or high-fat high-sucrose (HFHS, *N* = 5). Means +/- SEM are plotted. *p < 0.05, **p < 0.01, ***p < 0.001, ****p < 0.0001 by ANOVA for parametric data or Kruskal-Wallis if not.

To determine the respective impact of fat *vs*. sugar on the immune system, we first performed cytometry analysis on the spleen and MLNs of mice. Interestingly, we observed that all studied immune cells population were decreased in MLNs of HFHS-fed mice, whereas they were increased in HF-fed mice compared to NC-fed mice (Figures 2A–F). Specifically, numbers of leukocytes, myeloid cells, and lymphocytes, including B and T cells, were decreased in HFHS-fed mice, whereas they were increased in HF-fed mice compared to NC-fed mice (Figures 2A–F). However, these cells were not significantly disturbed in the spleen (Figures 2G–K) except myeloid cells representing 8.5% of leukocytes in HFHS-fed mice, while they only represented 2.1% in NC-fed mice (Figure 2H, Supplementary Figure 2A). Likewise, T-cells represented 25.9% of lymphocytes in HFHS-fed mice vs. 31.9% in control animals (Figure 2J, Supplementary Figure 2A). Among the lymphocytes in the MLNs, while NK cells amounted to an average of 18.7% CD45+CD3- cells in NC-fed mice, they expanded to 24.6% in HFHS-fed mice and 45.8% in HFD-fed mice (Supplementary Figure 2A). Likewise, T-cell subpopulations, including CD4+ and CD8+ cells, were strongly decreased in HFHS-fed mice MLNs but not significantly in the spleen, compared to control animals (Figures 2L–O). Significantly, Th17 cells were strongly decreased in MLNs of HFHS-mice and slightly increased in the spleen than NC-fed animals (Figures 2P,U).

We next focused on HFHS-fed mice, and to further dissect the local and systemic immune response, we measured cytokine levels in plasma and colonic tissues. As expected, cytokines levels in plasma (Supplementary Figures 2B–D) were significantly increased in DSS-treated mice fed with NC compared to untreated animals (IL-1β: +25.39 pg/mL, *p* = 0.0212; IL-6: +77.34 pg/mL, *p* = 0.0005; TNF-α: +108.8 pg/mL, *p* = 0.0351). Unexpectedly, these cytokines were not increased in plasma of colitic HFHS-fed mice compared to not-treated HFHS-fed animals excepted for IL-6 (+75.56 pg/mL, *p* = 0.0002; Supplementary Figure 2C). Then we measured levels of several chemokines in the colonic tissues of HFHS-fed mice. In contrast to plasma, chemokine levels were higher in colonic tissues of HFHS-fed colitic mice compared to DSS-untreated animals (RANTES: +295.0 pg/mL, *p* = 0.015; Eotaxins: +760.7 pg/mL, *p* = 0.037 and KC: +55.67 pg/mL, *p* = 0.026; Supplementary Figures 2E–G).

In summary, these findings indicate that fat and sugar induced similar intestinal lesions but have a differential effect on the immune system. In contrast to HF that increased immune cells, sucrose enrichment decreases the number of immune cells in both MLNs and the spleen. While HFHS causes impaired systemic cytokine response, chemokines recruitment is maintained in the colon of these animals.

### High-Fat High-Sucrose Diet Induces an Overall Down-Regulation of the Colonic Transcriptome of Healthy Mice

**Figure 3:**
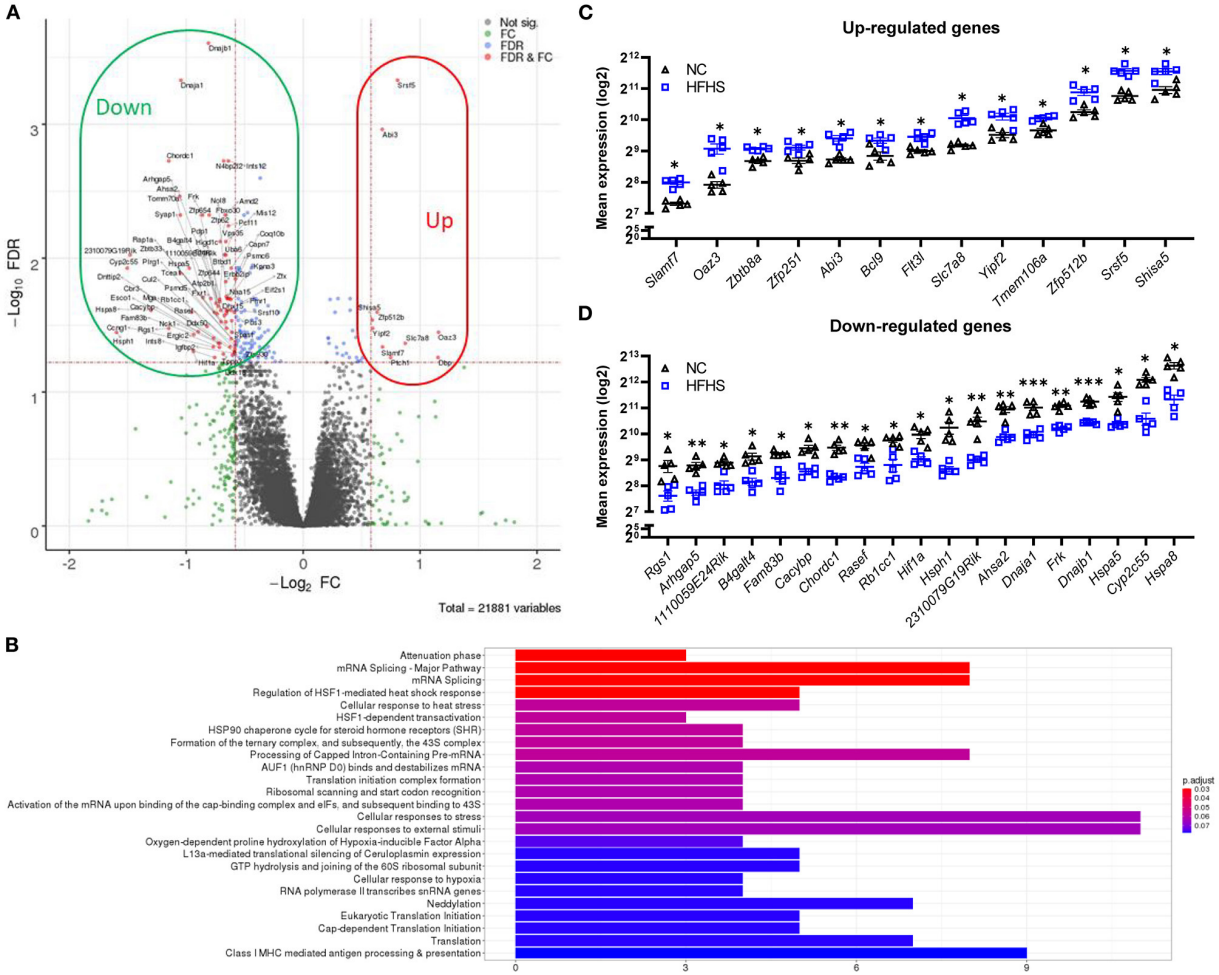
High-fat high-sucrose diet triggers multiple cellular stress responses on the colonic transcriptome. **(A)** Volcano plot and **(B)** Top Reactome pathways of dysregulated genes for HFHS-fed mice (*N* = 5) vs. NC-fed (*N* = 5). Statistical analyses identified 154 differentially expressed genes, 86% (132) being under-expressed in HFHS-fed compared to NC-fed mice (FDR < 0.05; fold change > 30%). **(C, D)** Top dysregulated genes in mice fed a HFHS diet compared with NC diet. Mean expression values (log2) are plotted with standard deviations for up- and down-regulated genes. FDR * ≤ 0.05, ** < 0.01, *** < 0.001.

To better understand how HFHS may predispose to disease risk in later life, transcriptomic analyses were performed on colonic tissues of DSS-untreated mice. Interestingly, in healthy mice, HFHS caused a strong gene down-regulation compared to NC-fed mice (Figure 3A). Statistical analyses identified 154 differentially expressed genes, 86% (132) being under-expressed in HFHS-fed mice (FDR <0.05; fold change > 30%) (Supplementary Dataset 1). These genes were predominantly involved in response to diverse cellular stresses such as HSF1-mediated heat-shock, external stimuli, unfolded protein stress *via* HSP90 chaperone complex, and to a lesser extent in the regulation of transcription and translation, as well as in Class I MHC mediated antigen processing and presentation (Figure 3B). Functional annotations with OpenTargets confirmed an association between the HFHS gene signature and nutritional and metabolic diseases such as diabetes (62 targets), abnormalities of the immune system (75 targets), and infectious diseases (104 targets). Interestingly, they also unraveled an association between HFHS and “colitis” (37 targets) or IBD (47 targets) despite the absence of DSS treatment, which is consistent with the pre-IBD state observed at the endoscopic and histologic levels (all *p-*values < 0.001) (Supplementary Table 4). Among the upregulated genes in HFHS-fed mice, we observed *Shisa5*, which encodes an endoplasmic reticulum protein involved in the apoptotic process (+1.5-fold; FDR = 0.029) and *Slamf7*, involved in cell-cell interactions by modulating the activation and differentiation of a wide variety of immune cells and in response to anti-TNF therapy in IBD (24, 25) (+1.6-fold; FDR = 0.046). Conversely, *Rgs1*, which encodes for a protein inhibiting B cells chemotaxis, was strongly under-expressed (−2.22-fold; FDR = 0.033) in HFHS-fed mice (Figure 3C), while being overexpressed in active IBD (26). Stress-related genes were under-expressed in HFHS-fed mice: *Hsph1*, which encodes a member of the heat shock protein 70 family (−3-fold; FDR = 0.036), *Hspa8* (−2.5-fold; FDR = 0.024), *Hspa5* (−2.1-fold; FDR = 0.013), and *Rb1cc*, indirectly implicated in autophagy *via* Atg16L1 interaction (−1.9-fold; FDR = 0.025), all coding for heat shock proteins (HSP) involved in autophagy and endoplasmic reticulum stress. Moreover, chaperones associated with these HSP were also downregulated in HFHS-fed mice: *Dnajb1* (−1.8-fold; FDR = 0.0002) and *Dnaja1* (−2.1-fold; FDR = 0.0005), both implicated in the regulation of heat shock proteins, or *Chordc1*, encoding a protein proposed to act as co-chaperone for HSP90 (−2.2-fold; FDR = 0.003) and *Ahsa2, a* pseudogene proposed as an HSP90 stimulating co-chaperone (−2.2-fold; FDR = 0.003) (Figure 3D). Overall, HFHS modulated several molecular pathways involved in cellular stress-related pathways in the healthy intestine of mice.

### High-Fat High-Sucrose Induces Dramatic Changes in Gut Microbiota and Its Derived Metabolites in Healthy Mice

**Figure 4:**
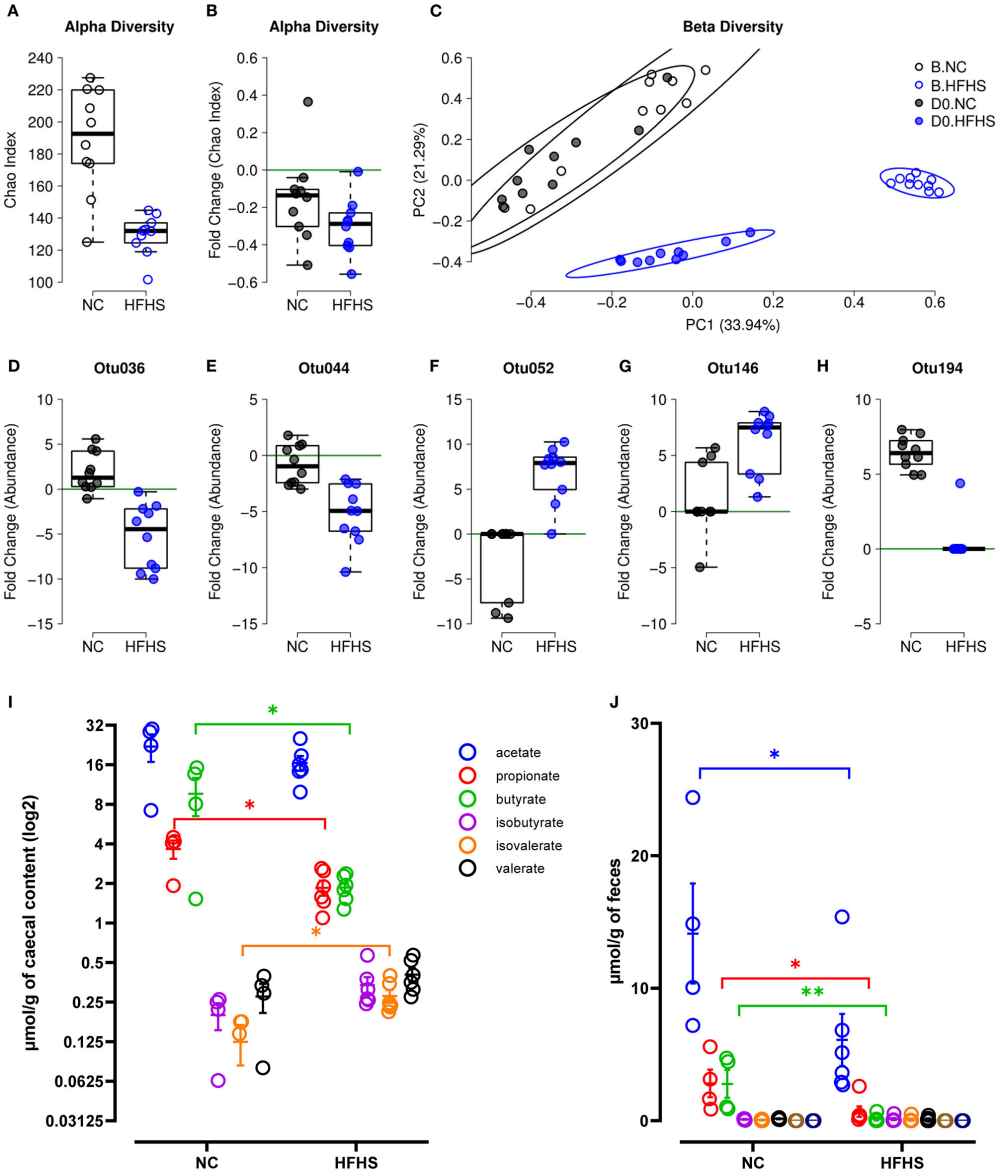
High-fat high-sucrose diet induces gut microbiota dysbiosis. **(A)** α-diversity measured by Chao index at baseline B for mice fed with normal chow (NC, *N* = 10) vs. high-fat high-sucrose diet (HFHS, *N* = 9). **(B)** Evolution of species richness measured by a fold change of Chao index at D0 relative to B. **(C)** PCoA of Bray-Curtis β-diversity index showing no NC-fed vs. HFHS-fed mice, at B and at D0. **(D–H)** Fold change of indicator OTU's abundances at D0 relative to B. **(I, J)** Short Chain Fatty Acids concentrations in μmol/g of caecal content or feces of NC-fed or HFHS-fed mice. Means +/– SEM are plotted. *p < 0.05, **p < 0.01 by ANOVA for parametric data or Kruskal-Wallis if not when more than 2 groups are compared, Mann-Whitney test or *t*-test for 2 groups comparison.

Prior to colitis, we investigated the impact of diet on the fecal microbiota by comparing its diversity and composition before starting the different diets (baseline or B) and after eight weeks of dietary intervention (day zero or D0). We observed that HFHS-fed mice had a lower α-diversity at baseline for the Chao (Figure 4A) and Shannon (Supplementary Figure 3A) indices because of the acclimatization period (diet was not the same throughout the acclimatization period and the experiment). To evaluate the diet's impact beyond the initial founder effect, we calculated a fold change of the α-diversity indices at D0 relative to baseline. We observed no significant difference between each diet group's fold changes, both for Chao (Figure 4B) and Shannon indices (Supplementary Figure 3B). These results indicated that the dietary intervention did not strongly influence the α-diversity of the intestinal microbiota. Considering β-diversity, we observed a strong association with the diet for each time point (Supplementary Table 5), and for both Bray-Curtis and Jaccard indices (Figure 4C, Supplementary Figure 3C), with explained variance >42%. However, within diet groups, a significant difference between time points was observed only for HFHS-fed mice, with explained variance >25% for Jaccard index and >71% for Bray-Curtis index (Supplementary Table 5). These results indicate that despite the initial difference between animal groups, the HFHS-fed mice had an essential change in the composition of their fecal microbiota, strongly affecting core abundant bacteria, and to a lesser extent, the prevalence of less abundant species. Consistently, we identified five Operational Taxonomic Units (OTUs) indicators for diet, with OTUs defined as follows: i) abundance/prevalence is significantly associated with diet at D0 but not at baseline, and ii) fold change between D0 and B is significantly associated with diet (Supplementary Figure 7). Precisely, two OTUs belonging to the order Clostridiales (Otu036, Otu044) showed an apparent decrease in their relative abundance in HFHS-fed mice while remaining at a similar abundance in NC-fed mice (Figures 4D,E, Supplementary Figure 3). Two OTUs belonging to the family Lachnospiraceae (Otu052, Otu146) showed a strong increase of their relative abundance and prevalence in HFHS-fed mice (Figures 4F,G, Supplementary Figure 3, Supplementary Table 5). One OTU belonging to the genus *Barnesiella* (OTU194) was not found at baseline but was ubiquitous at D0 in NC-fed mice, while present in only one HFHS-fed animal, in lower abundance (Figure 4H, Supplementary Figures 3P–U). These results suggest that HFHS can significantly impact individual bacteria, most of them belonging to Firmicutes, known to produce short-chain fatty acids (SCFAs) (27) (Supplementary Figure 7). These biomarkers represent together more than 90% of the OTUs, while Firmicutes represent ~50% of the entire community, indicating that bacteria belonging to this phylum might be significantly impacted by the dietary intervention.

We next measured SCFAs concentration in caecal contents and feces of mice, and showed that microbial changes induced by HFHS were followed by a significant decrease in caecal and fecal SCFAs concentrations (Figures 4I,J). Indeed, caecal propionate and butyrate concentrations were decreased, whereas isovalerate concentration increased in HFHS-fed mice compared to control animals (*p* < 0,05) (Figure 4I). Likewise, fecal acetate, propionate, and especially butyrate concentrations were profoundly reduced in HFHS-fed mice compared to control animals (*p* < 0,05) (Figure 4J).

To highlight the role of the gut microbiota in this experimental context, we performed fecal microbiota transplantation (FMT) in germ-free (GF) mice before inducing DSS-colitis (Supplementary Figure 4A). Despite the initial difference in GF-mice bodyweight before FMT, those who received microbiota from HFHS-fed mice recovered their initial weight after transplantation, contrary to NC-fed mice transplanted (Supplementary Figure 4B). Then, GF-mice that received microbiota from HFHS or HF-fed mice developed higher colitis than mice transplanted with fecal microbiota from control mice, as illustrated by higher bodyweight loss (Supplementary Figure 4D), survival rate (Supplementary Figure 4C). Importantly, at day 8, GF-mice that received HFHS-fed microbiota had an increased DAI (Supplementary Figure 4E).

### Incomplete Recovery of High-Fat High-Sucrose-Induced Damage Upon Cessation of Overconsumption

**Figure 5:**
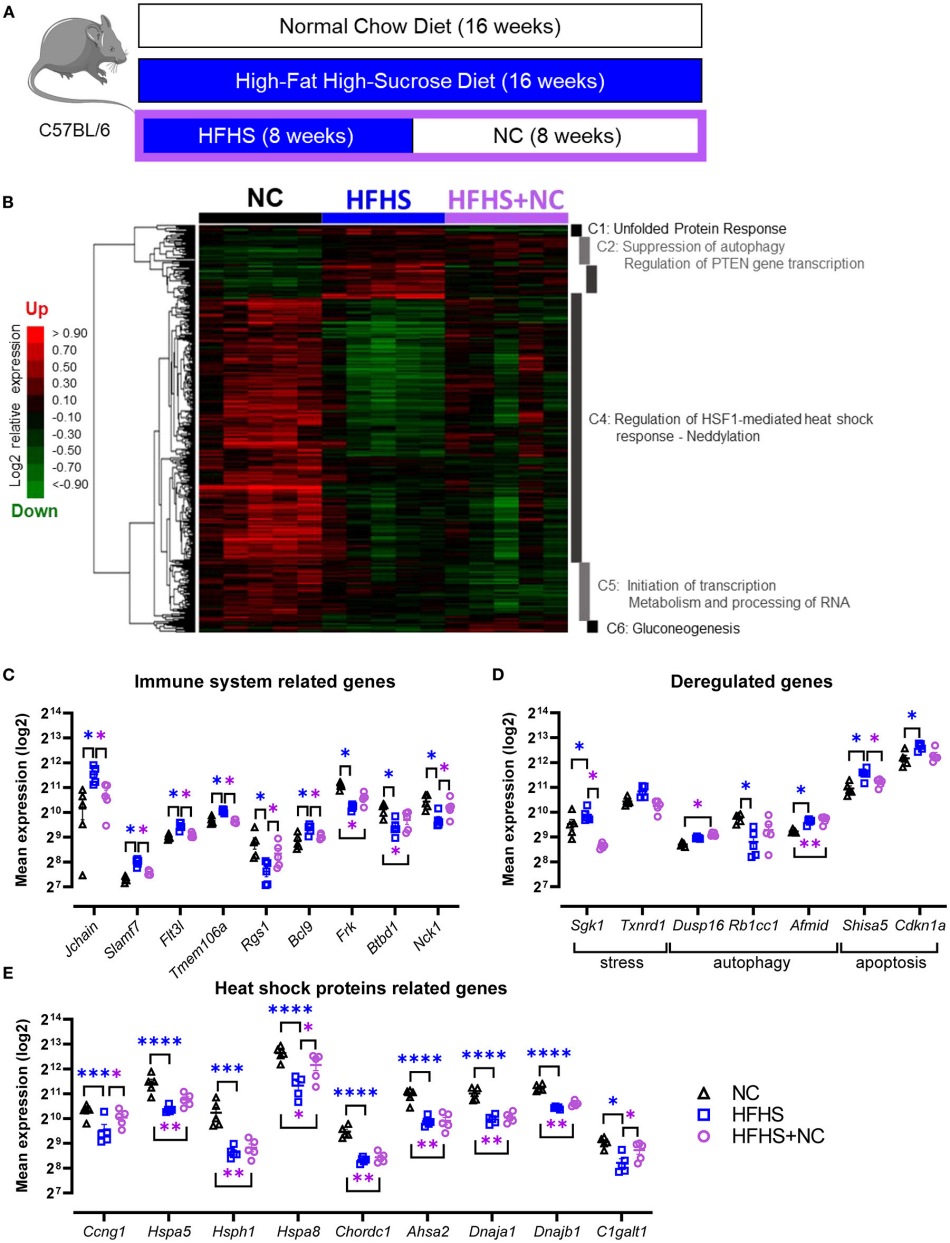
Partially reversible effects of high-fat high-sucrose diet on the colonic transcriptome. **(A)** Experimental design. Mice were fed with a normal chow diet (NC, *N* = 10) or a high-fat high-sucrose diet (HFHS, *N* = 10) for 16 weeks or 8 weeks and then switched back to NC for 8 weeks (HFHS+NC, *N* = 10). **(B)** Hierarchical clustering heatmap of the most variable genes in NC vs. HFHS vs. HFHS+NC fed mice. **(C–E)** Expression levels of relevant genes in clusters C1 to C6 for groups of mice fed with NC, HFHS or HFHS+NC, mean expression values (log2) are plotted with standard deviations for up- and down-regulated genes. FDR * ≤ 0.05, ** < 0.01, *** < 0.001, **** < 0.001.

To assess whether the changes induced by HFHS could be restored, we analyzed the gut microbiome and transcriptome of mice fed eight weeks with NC after eight weeks of HFHS (Figure 5A). Colonic transcriptome reprogramming detected in HFHS-fed mice was only partially reversible in HFHS+NC-fed mice (Figure 5B). Detailed results are described in Supplementary Material. Briefly, further hierarchical clustering identified six dysregulated signatures in HFHS+NC-fed mice, predominantly involved in unfolded protein response (UPR), autophagy, heat shock protein response, and metabolic pathways that play a crucial role in intestinal homeostasis (Figure 5B, Supplementary Table 6). Among upregulated genes in HFHS, many genes involved in p53/TP53-dependent apoptosis returned to regular expression levels (as observed in NC-fed mice) after switching diet while many HSP-related genes were down-regulated in HFHS-fed mice failed to recover their baseline expression in HFHS+NC-fed mice (Figures 5C–E). Regarding disease-associated targets, genes from clusters 2 and 5 were still associated with immune system diseases (75 targets) and gastrointestinal diseases (60 targets) including IBD (27 targets), ulcerative colitis (20 targets), Crohn's disease (15), “colitis” (22 targets), and digestive system infectious diseases (44 targets) (all *p-*values < 0.09; Supplementary Table 7).

**Figure 6:**
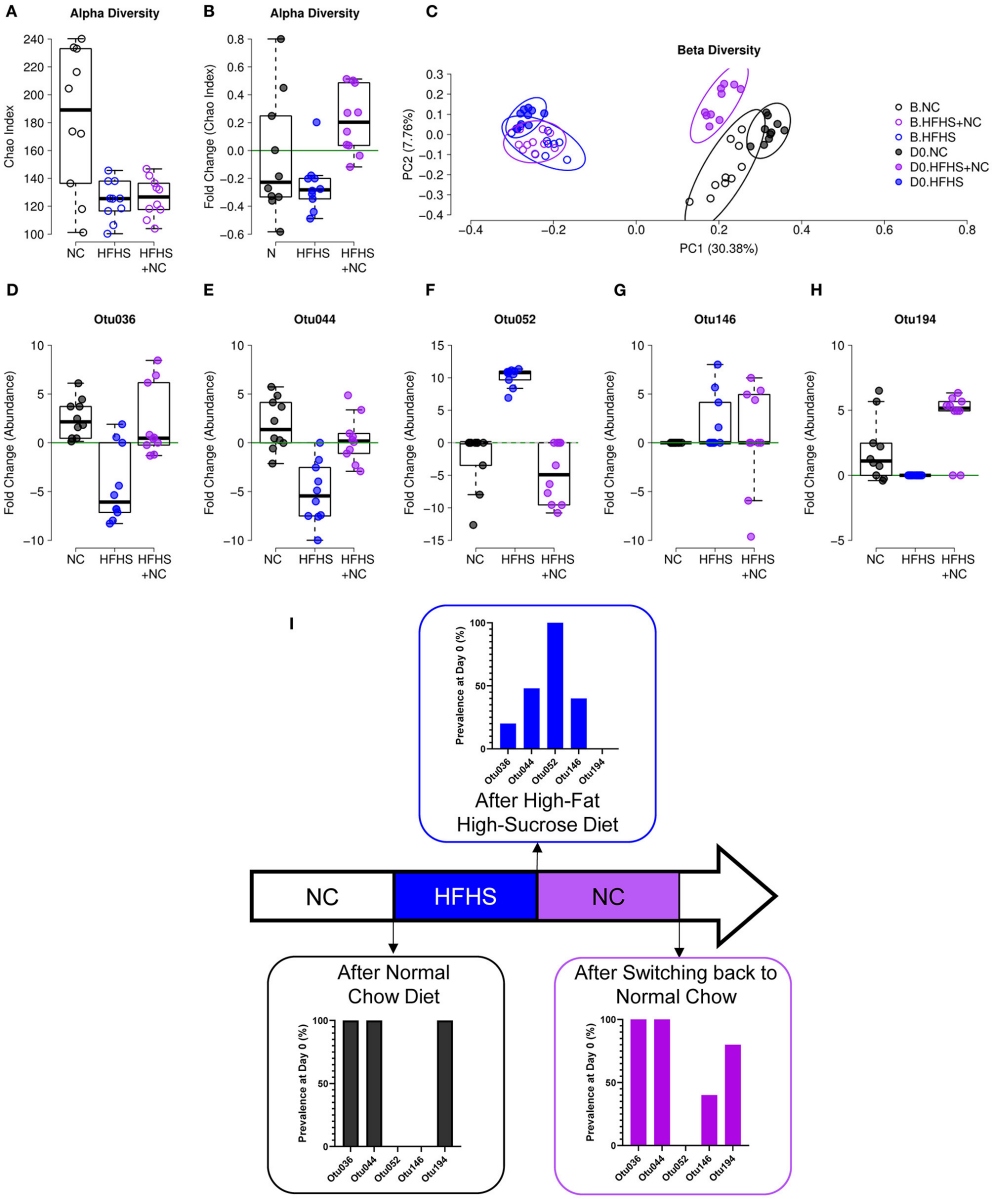
Incomplete recovery of microbiota dysbiosis. **(A)** α-diversity measured by Chao index of NC-fed, HFHS-fed, or HFHS+NC-fed (*N* = 10 per group) mice at baseline B. **(B)** Evolution of species richness measured by a fold change of Chao index at day zero (D0) relative to B. **(C)** Principal coordinate analysis (PCoA) of Jaccard β-diversity index showing NC-fed, HFHS-fed, or HFHS+NC-fed mice, at B and D0. **(D–H)** Fold change of abundances at D0 relative to B of the five operational taxonomic units (OTUs) identified as an indicator for diet in the 8-weeks experiment (see Supplementary Figure 6 and EV8). **(I)** Prevalence of indicated OTUs at D0 after 16 weeks of NC, HFHS, or NC following HFHS.

Similar observations were made on the composition of the gut microbiota. Detailed results are described in Supplementary Material. Briefly, NC-fed mice did not vary in α-diversity, while HFHS-fed mice had a lower diversity at D0 compared to baseline, and the HFHS+NC-fed group had a higher diversity at D0 compared to baseline only for the Chao index (Figures 6A,B). Regarding β-diversity, all groups were significantly different from each other at D0, but the HFHS-fed mice showed the highest distance to the other groups (Figure 6C). Among the indicators identified in the eight-week protocol, Otu036, Otu052, and Otu194 showed an interesting pattern, where the HFHS+NC-fed mice were in line with NC-fed mice and opposed to HFHS-fed mice, despite an initial difference according to diet group (Figures 6D–I, Supplementary Figure 7).

These results indicate that (i) HFHS has a consistent effect on microbial composition, given that four out of five biomarkers showed a similar pattern in both protocols, and (ii) a return to NC diet after eight weeks of HFHS can restore microbial abundances for four out of five diet-specific species identified in the eight-week protocol. Consistently, colitis severity was improved in HFHS-fed mice (Supplementary Figure 6).

Taken together, these results suggest that the long-term harmful effects of HFHS on gut microbiota and transcriptome can be partially corrected even after the return to a normal chow: some transcriptomic imprints lasted, and some effects persisted after exposure to HFHS on the gut microbiota.

## Discussion

Dietary patterns are important environmental triggers in IBD (6, 28). Overconsumption of dietary sugar has been linked to a rise of several non-communicable diseases (2–5). Recently, hyperglycemia was shown to disrupt the intestinal barrier and promote risk for enteric infections (11), while a high-sugar diet (at very high dose 55% sucrose) was reported to enhance the susceptibility to colitis by depletion of SCFAs after only two days of dietary intervention (12). Likewise, short-term intake (seven days) of a low dose of sugar (10% glucose or fructose in drinking water) did not trigger inflammatory responses in a healthy gut but markedly altered gut microbiota composition, leading to a predisposition to colitis (13). Here, we first confirmed that moderate doses of sucrose and fat (30% sucrose 25% fat) aggravates experimental colitis (12, 13). The DSS-colitis model induced profound intestinal damage and immunological disruption (29). Despite the growing interest of the scientific and medical community, the direct effects of long-term high-fat-high-sugar intake on healthy individuals' gut and the potential reversibility of those effects remain poorly described.

Therefore, we explored the long-term impact of additive effects of fat and sugar on intestinal homeostasis and focused on the specific effects of such diet on healthy mice intestine. Thaiss *et al*. demonstrated that glucose induced barrier alterations *in vitro* and increased permeability in diabetic mice (11). Unexpectedly, we showed that endoscopic lesions were observed in long-term HFHS-fed mice not treated with DSS (Figures 1F,G), whereas Khan *et al*. reported that short-term exposure to sugar minimally affected gut physiology (13). In our model, we can explain the lack of impact of HFHS on gut permeability (assessed by FITC-dextran, Supplementary Figure 1D) by the moderate hyperglycemia in mice (blood glucose average 2.5 g/L (Figure 1C); which is in agreement with glycemia observed in overweight C57BL/6 mice) while in diabetic models blood glucose exceeds 4.5 g/L (30).

To clarify the contribution of fats in our model, we used a HF diet with moderate dose. Indeed, HF is known to promote inflammation and exacerbates colitis severity at a very high dose (60% kcal from fats) (31). In our study, fats represented 25% of the regimen and, as expected, did not significantly influence the bodyweight or insulin/glucose sensitivity (Figures 1D,E) (32). More importantly, a 25%-HF did not influence colitis severity in our model so we can conclude that it's the addition of 30% sucrose in the HFHS diet that is responsible for the effects observed.

Overweight-induced hyperglycemia has been shown to impair immune defenses, as illustrated by diabetes-induced immunodepression that increases susceptibility to infections (33, 34). The relationships between hyperglycemia and pathological inflammation have been extensively studied (35). Our flow cytometry data showed (Figure 2, Supplementary Figure 2) a differential effect of fat *vs*. sugar on immune cell populations of MLNs and spleen. While HF increased the whole immune cell population in MLNs of mice, HFHS caused the opposite effect and especially strongly decreased the number of T cells in both spleen and MLNs of mice. Consistently, we observed a broadly depressed cytokine profile of HFHS-fed mice (Supplementary Figure 2), which may explain the susceptibility to enteric infection observed in hyperglycemic mice (8, 11). It would have been intersting to determine cytokine profile in HF-fed mice compared to HFHS-mice it is one of the limitations of our study. However, it can be assumed from other studies that HF diet increased pro-inflammatory cytokines secretion (36). Considering that such impaired immune defenses are a hallmark of IBD, this strengthens the link between sugar homeostasis and intestinal inflammation (37).

Furthermore, enlarged spleen and increased splenic myeloid and Th17 cells were observed in HFHS-fed mice, whereas they were decreased in MLNs, suggesting that immune cells may be mobilized on the inflammation site, i.e., the colon, similar to what has been observed with HF-induced colonic dysbiosis (38). It would have been necessary to explore this hypothesis by performing IHC stainings of flow cytometry on colons samples, it is one of the limitations of our study. Consistently, at the histological level, we observed immune cell infiltration in the colon of HFHS-fed mice (Supplementary Tables 2, 3). A recent study underpins a pro-inflammatory role for dietary sugar in LPS-stimulated mononuclear phagocytes, which occurs at the expense of metabolic flexibility (15), but further studies are needed to understand how metabolic reprogramming of several immune cell types can influence the functionality of immune cells (39).

Transcriptomics changes induced by sugar overconsumption in a healthy intestine were unknown. Our transcriptomic results showed that HFHS might predispose intestinal mucosa to IBD state. An overall down-regulation of the transcriptome was observed in HFHS-fed mice (Figure 3). Functional annotations reported in HFHS-fed mice not subjected to DSS treatment were significantly associated with IBD (Crohn's disease, ulcerative colitis, and “colitis”), confirming the observed endoscopic pre-IBD state. Immune reactivity to heat-shock-protein (HSP) resulted from inflammation in various disease animal and human inflammatory conditions such as diabetes (40). This is in line with previous reports looking at small bowel in a cancer mice model (41). In the colonic mucosa of HFHS-fed mice, *Hsph1, Hspa8*, and *Hspa5*, involved in autophagy and endoplasmic reticulum stress, were strongly under-expressed. The same observation was made for some chaperones implicated in HSP regulation, such as *Dnajb1, Chordc1*, and *Ahsa2*. However, among the limitations of our study, it would have been interesting to further explored (and to confirmed transcriptional observations) the potential mechanism for increased susceptibility to DSS-induced inflammation in HFHS-fed mice by IHC stainings and/or Western-blotting analysis on colonic samples.

Particularly, many of these HSP are ubiquitous and involved in cellular homeostasis maintenance under stress conditions (e.g., ATP hydrolysis, protein folding) (40). Importantly, we explored the reversibility of HFHS-induced alterations by switching mice back to NC diet after eight weeks of HFHS (Figure 5). We showed that only a small part of the transcriptome could be restored, leaving an entire pan of the program dysregulated. This could predispose or lower the tolerance of the intestinal mucosa to injury. For example, concerning HSP-related genes, only 3 genes (Ccng1, Hspa8, and C1galt1) recovered the same expression level in HFHS+NC-fed mice (i.e., the expression level of NC-fed mice) and were decreased in HFHS-fed mice (Figure 5). Furthermore, small gene clusters were enriched with CEBPB targets (CCAAT/enhancer-binding proteins, essential for adipogenesis and gluconeogenesis and highly expressed in adipose tissue) in HFHS-fed mice, which regulate the main functions of the inflammatory response, intrinsically linking the stress response to carbohydrate homeostasis (42). Since HFHS decreases energy needs, the increase in endoplasmic reticulum stress should be viewed as a way to counterbalance the downregulation of these transcription factors related to HSP (43), further contributing to the pre-IBD state. In addition to the diet-induced damage and disturbances described in this study, we observed the dysregulation of carcinogenesis-related genes in the colonic mucosa of HFHS-fed mice. This observation aligns with increasing evidence that dietary sugars are involved in colorectal carcinogenesis, both in humans and animals (16, 37, 44). However, this was outside the scope of our study, and the links between HFHS, colitis, and colorectal cancer will require further investigation.

Finally, given the growing body of evidence linking microbial dysbiosis to IBD (45), we investigated the extent to whether intestinal bacteria may contribute to the effects described here. A previous report using short-time exposure to sugar (12) suggested that long-term diet exposure does not condition the DSS-related changes in α-diversity. However, we could observe a great difference in community composition between the diet groups (Figure 4). As expected, dietary intervention caused significant changes in animals' microbiota composition, both qualitatively and quantitatively (7, 46, 47), findings which were further supported by FMT experiments. In line with previous reports (12, 13), our results indicate that HFHS strongly impacts some bacterial taxa, most of them belonging to Firmicutes that are known to produce SCFAs and may enhance the epithelial permeability in response to high caloric diet (48, 49). This phylum is composed of gram-positive bacteria and includes the gut commensal *Clostridia*, which was shown to be a key player of intestinal homeostasis (50). Some members of the Clostridiales family were present in lower abundance in HFHS-fed mice not subjected to DSS-treatment while some *Lachnospiraceae* species increased, changes that were not observed with short-term exposure to sucrose (12). In addition to previous studies (12, 13) showing that a few days of sugar decreased only fecal and caecal acetate concentrations, we showed that 8-week overconsumption of sugar profoundly decreased both acetate, propionate, and butyrate concentrations. Our observations indicate that long-term overconsumption of sugar has a strong impact on intestinal microflora and their metabolites. SCFAs are known to play a key role in health maintenance by promoting both lipid, glucose, and immune homeostasis (51), so their decrease may also contribute to the observed pre-IBD state.

Even if additional work is needed to demonstrate that this may contribute to the pre-IBD state, it is noteworthy that the depletion of bacteria belonging to *Barnesiella* (Otu194) was observed in HFHS-fed mice while it was ubiquitously present in control animals (i.e., NC-fed mice). This may contribute both to the pre-IBD state, and the severity of DSS-induced colitis observed in this study. Indeed, given that an increase of *Barnesiella spp*. after oligosaccharide treatment was shown to decrease susceptibility to DSS-induced colitis in mice (17). Of interest, returning to the NC diet after 8-weeks of HFHS does not allow full recovery of this bacterial species (Figure 6). Similarly, the increase in *Lachnospiraceae* was only partially reversible, as Otu146 was absent in NC-fed mice but remained high in HFHS+NC-fed mice. Previous experiments (12, 13) demonstrated that sucrose induces very rapid changes in the microbiota composition. Our findings suggest that these modifications are persistent and difficult to reverse after long-term overconsumption of fat and sugar, even after returning to the NC diet for several weeks. Therefore, long-term sucrose and fat overconsumption induce some irreversible intestinal damage.

In summary, long-term overconsumption of fat and sugar induces a pre-IBD state under healthy conditions. This infraclinical state is characterized by decreased immune cell populations in MLNs and dysregulation of stress-related genes in the colonic mucosa leading to (i) gut microbiota dysbiosis, (ii) spontaneous endoscopic lesions, and (iii) global transcriptome alterations that were partially reversible. Overall, our results support observations in IBD patients that suggest a beneficial effect of reduced sugar consumption (10, 18, 22, 52). However, to ensure that the results observed in this study are translaTable to humans, intervention trials need to be initiated. In this regard, the increased consumption of sugars in the general population is alarming (53), highlighting the urgent need to follow WHO recommendations (3–5).

## Materials and Methods

### Animals and Diets

Male C57BL/6J mice were purchased from Janvier Labs (Le Genest-Saint-Isle, France). All procedures were performed following guidelines established by the European Convention for the protection of Laboratory Animals (54) and with a project approval (authorization N°APAFIS#16630) delivered by the French ministry of research. The animals were maintained on a strict 12-h light-dark cycle and were housed at 22–23°C, in cages containing a maximum of 5 animals, with ad libitum access to food and water. All animal experiments were repeated at least two times on two separate occasions. Before dietary interventions, mice were randomized to ensure that no incidental pre-diet differences in body weight existed between the different groups. Mice were fed with a High-fat high-sucrose Diet (HFHS; U8960 v5, Safe Diets, Augy, France), High Fat diet (HF U8960 v143, Safe Diets, Augy, France), or Normal Chow diet (NC; A04, Safe Diets, Augy, France) for 8 or 16 weeks (Figure 1A).

### Induction of Colitis

For each dietary regimen (NC, HFHS, or HF), half of the animals were treated with DSS to induce colitis after eight weeks of diet (Figure 1A). Mice were continued on the same diet during colitis, induced by administration of 3% dextran sulfate sodium (DSS, molecular weight 36 000–50 000, MP Biomedicals, Strasbourg, France) dissolved in drinking water for five days. DSS solution was replaced thereafter by normal drinking water for another five days as previously described (55). Mice were sacrificed on the 10th day or upon reached the endpoint (i. e. > 20% weight loss).

### Fecal Microbiota Transplantation

Germ-Free (GF) C57BL/6J mice were housed at gnotobiotic facilities at the Hannover Medical School (Hannover, Germany), and Fecal Microbiota Transplantation (FMT) experiments were performed at Technical University Dresden (Dresden, Germany). For microbial reconstitution of GF mice, 8-week-old GF C57BL/6J mice received fecal material obtained from individually WT C57BL/6J HFHS-fed or HF-fed or NC-fed mice. Stool pellets were freshly dissolved in PBS with 0.05% L-cysteine hydrochloride (Sigma-Aldrich; 4 pellets per 5 ml of PBS), and mice received 100 μl solution by gavage. After one week, colitis was induced by administration of 2% DSS dissolved in drinking water for five days. DSS solution was replaced thereafter by normal drinking water for another three days.

### Collection of Samples

For gut microbiota analysis, fresh stool samples from isolated mice were collected in tubes, immediately frozen in liquid nitrogen upon collection, and stored at −80°C until DNA isolation. Blood was collected by submandibular bleeding (56) in heparinized tubes (Microtainer® BD medical, Franklin Lakes, NJ), and the plasma was obtained by centrifugation at 2000 xg > 3min at room temperature. The entire colon was removed from the caecum to the anus. The colon length was measured, then washed with phosphate-buffered saline PBS 1X to remove the remaining content. The spleen was removed and weighed as previously described (57). Samples from the colon were taken, divided into 0.5 cm long pieces, snap-frozen in liquid nitrogen, and stored at −80°C until use.

### *In vivo* Metabolic Tests

Metabolic tests were performed with 10 mice per group using standard procedures (58). Oral glucose tolerance tests (OGTT) were performed with mice fasted overnight for 6 h. Blood glucose concentrations were determined before and after administering glucose solution orally via gavage (2g glucose/kg). Blood glucose levels were determined at defined post-gavage time points by analyzing blood obtained from the tail vein with a portable glucometer (Glucometer OneTouch® Verio Reflect, LifeScan Europe GmbH, Zug, Switzerland). For insulin tolerance tests (ITT), mice were fasted for 3 h and then injected with human insulin (0.75 U/kg i.p.; NovoRapid®, NovoNordisk A/S, Denmark). Blood glucose levels were monitored as described for the OGTT assay.

### Measurement of the Colonic Epithelial Barrier Permeability by FITC-Dextran

On the day of the assay (day 5 of DSS-treatment), 4 kDa fluorescein isothiocyanate (FITC)-dextran (Sigma-Aldrich, St. Louis, MO) was dissolved in PBS 1X to obtain a stock solution of 80 mg/mL. Mice fasted for 4 h before gavage with 60 mg/100g. Blood was collected 3 h following gavage on heparinized tubes (Microtainer® BD medical, Franklin Lakes, NJ). FITC-Dextran was quantified in plasma by fluorescence (γ Ex/Em 485 nm/535 nm, ID3, Molecular devices) as previously described (11).

### Assessment of Disease Activity Index

Mice were daily weighed and evaluated for clinical symptoms. The Disease Activity Index (DAI) was determined on a scale from 0 to 4 and calculated as the mean of three individual subscores (body weight loss, stool consistency, and blood in the stool) as previously described (59).

### Endoscopic Assessment and Scoring

Coloscopy was performed on the last day of the study (day 10), just before the mice were sacrificed. Mice were anesthetized by isoflurane inhalation. Distal colon and rectum were examined using a rigid Storz Hopkins II mini-endoscope (Storz, Tuttlingen, Germany) coupled to a basic Coloview system (with a xenon 175 light source and an Endovision SLB Telecam; Storz). All images were displayed on a computer monitor and recorded with video capture software (Studio Movie Board Plus from Pinnacle, Menlo Park, CA). Three independent trained-readers performed a blind determination of endoscopic scores. Ulcerative colitis endoscopic index of severity (UCEIS) was calculated as the sum of three subscores (vascular pattern, bleeding, and erosions/ulcers), as previously described (60). The final grade was defined as the mean of the three independent assessments.

### Histological Assessment and Scoring

Colon samples were extensively washed and fixed with 4% paraformaldehyde for 24 h under agitation. Samples were washed, paraffin-embedded, and sectioned. Colitis was histologically assessed on 5 μm sections stained with hematoxylin-eosin-saffron (HES) stain. The histological colitis score was calculated blindly by expert pathologists, as previously described (61). Briefly, disease scoring based on six histological features: acute inflammatory cell infiltrate (polymorphonuclear cells in the lamina propria), crypt abscesses, mucin depletion, surface epithelial integrity, chronic inflammatory cell infiltrate (round cells in the lamina propria), and crypt architectural irregularities. Each feature was graded on a 4-point scale corresponding to none, mild, moderate, or severe. The final grade was defined as the mean of the two independent assessments.

### Flow Cytometry

Mesenteric lymph nodes and spleen samples were washed and conserved in complete RPMI (10 % FCS). Samples were first grinded on a nylon mesh (40 μm cell strainer, Greiner), and large debris was removed. Cell suspensions were filtered through a 70 μm mesh (Miltenyi Biotec) and centrifuged at 4 °C, 600 xg, for 5 min. Cell suspensions were then washed and resuspended in phosphate-buffered saline (PBS) supplemented with 2% bovine serum albumin (BSA) for counting on an automated cell before direct cell surface staining. For intracellular staining, cells were fixed and permeabilized with a commercially available fixation/permeabilization kit (eBioscience). Single-cell suspensions were stained with antibodies to the following markers: CD45-BV421, CD3-APC/Fire750, CD4-FITC, CD25-BV510, CD8a-BV711, CD335-PECy7, CD19-BV605, CD11b-PERcpCY5.5, RORγt-APC, and Foxp3-PE in presence of FCBlock CD16/32; all antibodies were obtained from Biolegend. The gating strategy is detailed in Supplementary Figure 8. Gallios cytometer (Beckman) was used for cell acquisition, and the flow cytometry data were analyzed with Kaluza software.

### Cytokine Bead Assay

Total protein was extracted from colonic tissue by lysing homogenized tissue with Bio-plex Cell lysis kit (Bio-rad #171304011, Bio-Rad Laboratories Inc, USA) and quantified by using the bicinchoninic acid assay method. All samples were normalized to 900 μg/mL for the assay. Cytokine levels in plasma and colonic lysates were measured using Bio-Plex Pro mouse cytokines 23-plex assay group I assay (Bio-Rad #M60009RDPD, Bio-Rad Laboratories Inc, USA), a BioPlex 200 instrument, and Milliplex Analyst software. Each biological replicate was assayed in technical duplicate. According to the manufacturer's instructions, protein concentrations were determined based on a standard curve described by the manufacturer-provided protocol and values for Lot #64212941.

### RNA Extraction and Microarray Experiment

Total RNA was extracted from 20 mg of colon sample using Trizol (Invitrogen, Carlsbad, California, USA) according to the manufacturer's recommendations. RNA was further digested with TurboTM DNase (Thermo Fisher, Waltham, Massachusetts, USA) and phenol-chloroform extracted. The quality of total RNAs was attested by O.D. 260 nm/O.D. 280 nm and O.D. 260 nm/O.D. 230 nm determination by spectrophotometry using a Nanodrop ND-1000 (NanoDrop Technologies; Wilmington, DE, USA) and using a 2,100 Bioanalyzer (Agilent Technologies; Massy, France). Samples with RNA integrity number > 8 were selected. RNA samples were aliquoted and stored at −80 °C, and later amplified and biotinylated using the Affymetrix GeneChip® WT PLUS reagent kit, then hybridized to Affymetrix Mouse Clariom S cartridges following the manufacturer's instructions. The arrays were washed and scanned according to the protocol GeneChip® Expression Wash, Stain and Scan for Cartridge Arrays.

### Transcriptomics

Fluorescence values corresponding to raw expression data for every 37 samples were extracted from CEL files using the R oligo package (https://bioconductor.org/) with the corresponding platform definitions (pd.clariom.s.mouse). Positive and negative control probes were removed, which left 21.881 probes, each corresponding to a unique and well-annotated gene. Quality control steps, data normalization, and unsupervised explorations were conducted as described previously (62). Corrections for batch effects between microarrays were performed with Combat (package sva from Bioconductor). Statistical analyses were achieved using linear modeling with empirical Bayes, p.values were computed by applying a moderated two-way *t*-test and adjusted for false discovery rate (FDR) following the Benjamini–Hochberg procedure. Hierarchical clustering heat maps were obtained on gene-median-centered data with uncentered correlation as a similarity metric. Volcano plots were rendered using EnhancedVolcano (Bioconductor). Functional annotations were performed with the OpenTargets platform (63) for disease associations and gene ontology enrichments and ReactomePA (64) for pathway analyses in mice. For all experiments, p.values < 0.001 or FDR < 0.05 indicated statistical significance, depending on the test availability and/or relevance.

### Microbiota Analysis

Bacterial profiling using 16S amplicon sequencing was performed as described previously (65). Briefly, DNA from fecal pellets was isolated using the PowerSoil DNA Isolation Kit (MoBio) according to the manufacturer's directions. Individual amplicons were tagged with specific multiplex identifier (MID) barcodes and pooled for library construction before sequencing. The 16S rRNA gene variable region V3-V4 was amplified and sequenced on an Illumina MiSeq with 2 × 250 bp. Raw sequences were first trimmed to remove bad quality tails and filtered for size using *CUTADAPT* (66) v1.1.4. Then *USEARCH* (67, 68) v10.0.240 was used to merge forward and reverse reads. Merged reads were filtered for size and expected error rate before dereplication and denoising, simultaneously removing chimeras. Reads present in less than two copies were filtered out as they likely represent PCR or sequencing errors. A custom *R* (69) script was used to transfer sequences into *MOTHUR* (70) v1.39.5, with which further processing was done according to the MiSeq SOP (71). Briefly, sequences were aligned to the *SILVA* (72) v132 reference database, classified against the *RDP* release 11 (trainset 16) (73) database, binned into operational taxonomic units (OTUs) at the species-level 97% identity threshold, and rarefied to 12,000 sequences per sample. A detailed account of the procedure is available in Supplementary Dataset 2. This procedure filtered 4 763 346 sequences for 147 samples, and lead 3 152 719 sequenced to be retained (66%) before subsampling (Supplementary Dataset 3). Additional formatting scripts are available in Dataset S4. The OTU Table obtained from MOTHUR was imported in *R* (69) for statistical analysis. *Alpha diversity* indices (Chao and Shannon) were calculated with *vegan* (74) package version 2.5–6. Fold changes between time points were calculated as: log2 (target value/reference value). Correlations between α-diversity indices or fold change and experimental parameters were tested using the Wilcoxon rank sum test. *Beta diversity* was evaluated with the *vegan* (74) package version 2.5–6, using the Bray-Curtis and Jaccard distances to build principal coordinate analysis (PCoA). Fold changes of OTU abundances between time points were calculated as: log2 [(target value+0.1)/ (reference value+0.1)]. The addition of 0.1 to both values avoids infinite values due to zero abundances, while keeping the ratios in the same trend. Correlations with experimental parameters were assessed with multivariate analysis of variance using package pairwiseAdonis (75). *Biomarkers* were found by correlating individual OTUs with experimental parameters with four complementary tests: Kruskal-Wallis rank sum test was done on OTU abundances and fold changes (i) or non-zero OTU abundances (ii); Pearson's chi-square test was performed on OTU prevalence (iii); indicator species analysis (iv) was conducted with package *indicspecies* (76). To evaluate the strength of association, Eta was calculated for Kruskal-Wallis tests and Cramer's V for chi-square tests. Where applicable, *p*-values were adjusted for multiple testing using the false discovery rate (“FDR”) correction. All necessary data and scripts for analysis are available in Supplementary Dataset 5.

### Evaluation of the Fermentative Activity of the Microbiota

The concentrations of short-chain fattyacids (SCFAs; i.e. acetate, propionate, butyrate, valerate, caproate, isobutyrate, isovalerate, and isocaproate) were measured in the cecal contents and stool samples of mice. Analyses were performed as previously described (77). Samples were water-extracted, and proteins were precipitated with phosphotungstic acid. Analyzes were performed by gas chromatography using a system (Autosystem; Perkin-Elmer, St. Quentin en Yvelines, France) equipped with a split/splitless injector, a flame ionization detector, and a capillary column (length, 15 m; inside diameter, 0.53 mm; film thickness, 0.5m) impregnated with SP100 (Nukol; Supelco, Saint-Quentin-Fallavier, France). The internal standard used was 2-ethylbutyrate. All samples were analyzed in duplicate. Data were collected, and peaks were integrated using Turbochrom software (Perkin-Elmer, Courtaboeuf, France).

### Statistical Analysis

All data are expressed as means ± SEM. Two-group comparisons were performed using Mann-Whitney *U-*test for non-parametric data or t-test for parametric data. ANOVA with Tukey's *post-hoc* test for parametric data or Kruskal Wallis with Dunn's correction for non-parametric data were used to compare more than two groups. *p* values < 0.05 were considered significant. Statistical details and the exact value of “N” can be found in the Figure legends. Statistical analyses were performed with GraphPad Prism 6 software (GraphPad Software, Inc, La Jolla, CA). All authors had access to the all data and have reviewed and approved the final manuscript.

## Data Availability Statement

The datasets presented in this study can be found in online repositories. The names of the repository/repositories and accession number (s) can be found in the article/Supplementary Material.

## Ethics Statement

The animal study was reviewed and approved by Comité National de Reflexion sur l'Ethique en Expérimentation animale CNREEA (authorization N◦APAFIS#16630 delivered by the French ministry of research).

## Author Contributions

DA performed the research, analysis and interpretation of data, and drafted the manuscript. MV performed microbial 16S analyses and provided microbiome expertise. SH performed bioinformatics and transcriptomic-related analyses. A-MA provided the histological data and histopathological expertise. CM performed SCFAs analysis. KP, AS, and SZ conducted the FMT experiments. DA, TK, CC, ND, MC, OB, and J-MA collected and/or analyzed the data. NN conducted the statistical analysis, DM and HL of flow cytometry. LP-B, TK, and FH were responsible for the conception and design of the study. LP-B and TK were responsible for interpretation of data, and drafting of the manuscript. All authors reviewed and approved the manuscript.

## Funding

This work was supported by Fondation pour la Recherche Médicale (FRM grant number ECO20170637494 to DA), by Conseil Scientifique de l'Université de Lorraine (AAP-003-212 to FH), by Association François Aupetit (AFA) and by the French PIA project ≪ Lorraine Université d'Excellence ≫ (ANR-15-IDEX-04-LUE to TK).

## Conflict of Interest

LP-B reports personal fees from AbbVie, Janssen, Genentech, Ferring, Tillots, Pharmacosmos, Celltrion, Takeda, Boerhinger Ingelheim, Pfizer, Index Pharmaceuticals, Sandoz, Celgene, Biogen, Samsung Bioepis, Alma, Sterna, Nestle, Enterome, Allergan, MSD, Roche, Arena, Gilead, Hikma, Amgen, BMS, Vifor, Norgine; Mylan, Lilly, Fresenius Kabi, Oppilan Pharma, Sublimity Therapeutics, Applied Molecular Transport, OSE Immunotherapeutics, Enthera, Theravance; grants from Abbvie, MSD, Takeda; stock options: CTMA. The remaining authors declare that the research was conducted in the absence of any commercial or financial relationships that could be construed as a potential conflict of interest.

## Publisher's Note

All claims expressed in this article are solely those of the authors and do not necessarily represent those of their affiliated organizations, or those of the publisher, the editors and the reviewers. Any product that may be evaluated in this article, or claim that may be made by its manufacturer, is not guaranteed or endorsed by the publisher.
