# Supplementary material for: Long-Term Overconsumption of Fat and Sugar Causes a Partially Reversible Pre-inflammatory Bowel Disease State
Source: Front Nutr. 2021 Nov 18;8:758518. doi: 10.3389/fnut.2021.758518 (PMC8637418; doi:10.3389/fnut.2021.758518)
Supplement: Supplementary file 2 [file Data_Sheet_2.docx]

Supplementary Material for Long-term overconsumption of fat and sugar causes a partially reversible pre-inflammatory bowel disease state

# Supplementary Results

## Gut microbiome and transcriptome alterations induced by high-fat high-sucrose diet are partially restored when returning to the normal chow diet

Colonic transcriptome reprogramming detected in HFHS-fed mice was only partially reversible in HFHS+NC-fed mice (**Figure 5 A**). Focusing on the dataset composed of NC, HFHS and HFHS+NC groups, 426 genes were tagged as most varying across samples (>10% of maximum variance, excluding outliers), which overlapped the top genes in HFHS vs NC (349 down- and 77 up-regulations). Further hierarchical clustering identified 6 dysregulated signatures in HFHS+NC-fed mice, predominantly involved in unfolded protein response (UPR), autophagy, heat shock protein response and metabolic pathways that play a key role in intestinal homeostasis (**Figure 5 B and Table S7**). Interestingly, UPR (cluster C1, increased in HFHS) and gluconeogenesis-related genes (cluster C6, decreased in HFHS) returned to their baseline expression levels in HFHS+NC-fed mice while suppression of autophagy (cluster C2, including genes such as *Dusp16, Rb1cc1* and *Afmid*) was still increased in HFHS+NC, and heat shock mediated response-related genes (cluster C4, decreased in HFHS) had not completely recovered their pre-HFHS levels after 8 weeks of NC in HFHS+NC-fed mice (**Figure 5 B**).

Among the upregulated genes in HFHS, were i) *Sgk1,* which encodes a serine/threonine protein kinase involved in response to cellular stress and ii) *Txnrd1,* which plays a key role in redox homoeostasis and is involved in regulation by PPAR-α pathway. These were conversely under-expressed in HFHS+NC-fed mice, by 2.4-fold and 1.5-fold respectively. Many genes involved in p53/TP53-dependent apoptosis also returned to normal expression (as observed in NC-fed mice) after switching diet, like *Shisa5* or *Cdkn1a*. Immune system related *Bcl9* (implicated in B-cell malignancies) almost returned to NC-like expression in HFHS+NC (**Figure 5 C**).

Many heat-shock-protein-related genes that were down-regulated in HFHS-fed mice failed to recover their baseline expression in HFHS+NC-fed mice. This was the case for *Hsph1,* *Hspa8, Hspa5,* *Chordc1* and *Ahsa2* Finally, expression of *C1galt1, which* encodes for the common core 1 O-glycan structure involved in Golgi dynamic, and precursor of many extended mucin-type O-glycan on cell surface and secreted glycoproteins, was 1.8-fold downregulated in HFHS-fed mice compared to control animals while returning to NC after HFHS seemed to correct expression of this gene but without reaching control levels. This was also the case for *Rgs1*, an immune system related gene involved in B cell chemotaxis inhibition (**Figure 5 C**).

Regarding at the microbiome, when comparing the fold changes of α-diversity indices between D0 (after 16 weeks of dietary intervention) and baseline, we observed that NC-fed mice did not vary in diversity, while HFHS-fed mice had a lower diversity at D0 compared to baseline, and the HFHS+NC-fed mice had a higher diversity at D0 compared to baseline for the Chao index, but not for the Shannon index (**Figure 6 B and S6 B and Table S8**). Consistently, we observed a strong association between diet and β-diversity at baseline, with NC-fed mice showing major differences when compared to the two other groups, with explained variance >36% for Jaccard index and >30% for Bray-Curtis index (**Figure 6 C and S6 C**).

Interestingly, at D0, all three groups were significantly different from each other, but the HFHS-fed mice showed the highest distance to the other groups, with explained variance >57% for Bray-Curtis and >47% for Jaccard, while the explained variance between NC-fed and HFHS+NC-fed mice was <38%, which is well in the range of the initial difference between the groups (**Table S8**). Moreover, within diet groups, we observed that the HFHS+NC-fed mice had the deepest change, with explained variance >44%, while for NC-fed mice the explained variance was <27%, and < 23% for HFHS-fed mice. These results indicate that returning to normal chow diet after exposure to HFHS can improve α and β-diversity by bringing HFHS+NC-fed mice back in the range of the NC-fed mice.

Consistently, we could identify 35 OTUs indicator for diet, defined as OTUs whose abundance/prevalence was significantly associated with diet at D0 but not at baseline, and whose fold change was also associated with diet (**Figure S6**). Among these 35 biomarkers, Otu044 was previously identified as an indicator for diet after eight weeks of HFHS (**Figure 6 D and Table S8**). This biomarker did not show a correlation with diet group at baseline, while its abundance and prevalence significantly decreased at D0 in HFHS-fed mice and remained sTable in NC-fed and HFHS+NC fed mice (**Figure 6 D and Figure S6 F, J, O, T**). Among the indicators identified in the eight-week protocol, Otu036, Otu052 and Otu194 showed an interesting pattern, where the HFHS+NC-fed mice were in line with NC-fed mice and opposed to HFHS-fed mice, despite an initial difference according to diet group (**Figure 6 E, G, I and Figure S6**). Finally, Otu146 was not associated with diet in this protocol (**Table S8**).

# Supplementary Tables

**Table S1. Endoscopic subscores.** NC: normal chow, HF: high-fat, HFHS: high-fat high-sucrose, DSS: dextran sulfate sodium.

|  | NC DSS^-^  (N = 18) | | HF DSS^-^ (N = 5) | | HFHS DSS^-^  (N = 20) | | NC DSS^+^  (N = 14) | | HF DSS^+^ (N = 5) | | HFHS DSS^+^ (N = 15) | |
| --- | --- | --- | --- | --- | --- | --- | --- | --- | --- | --- | --- | --- |
|  | Mean | SEM | Mean | SEM | Mean | SEM | Mean | SEM | Mean | SEM | Mean | SEM |
| Vascular Pattern | 1.0 | 0.0 | 1.7 | 0.2 | 1.4 | 0.1 | 2.2 | 0.2 | 1.8 | 0.3 | 2.3 | 0.2 |
| Bleeding | 1.4 | 0.2 | 2.2 | 0.4 | 2.1 | 0.2 | 2.2 | 0.2 | 2.4 | 0.3 | 2.7 | 0.2 |
| Erosions and Ulcers | 1.3 | 0.1 | 1.5 | 0.2 | 1.4 | 0.1 | 3.0 | 0.2 | 2.4 | 0.5 | 2.6 | 0.2 |
| TOTAL | 3.7 | 0.2 | 5.4 | 0.7 | 4.9 | 0.3 | 7.4 | 0.4 | 6.6 | 0.9 | 7.6 | 0.5 |

**Table S2. Histological subscores.** NC: normal chow, HF: high-fat, HFHS: high-fat high-sucrose, DSS: dextran sulfate sodium.

|  | NC DSS^-^  (N = 13) | | HF DSS^-^ (N = 5) | | HFHS DSS^-^  (N = 13) | | NC DSS^+^  (N = 13) | | HF DSS^+^ (N = 5) | | HFHS DSS^+^ (N = 12) | |
| --- | --- | --- | --- | --- | --- | --- | --- | --- | --- | --- | --- | --- |
|  | Mean | SEM | Mean | SEM | Mean | SEM | Mean | SEM | Mean | SEM | Mean | SEM |
| Acute inflammation | 0.0 | 0.0 | 0.0 | 0.0 | 0.0 | 0.0 | 1.000 | 0.277 | 0.4 | 0.245 | 1.833 | 0.207 |
| Chronic inflammation | 0.0 | 0.0 | 0.0 | 0.0 | 0.231 | 0.122 | 1.538 | 0.291 | 1.0 | 0.548 | 2.917 | 0.313 |
| Cryptic abscess | 0.0 | 0.0 | 0.0 | 0.0 | 0.0 | 0.0 | 0.154 | 0.104 | 0.0 | 0.0 | 0.0 | 0.0 |
| Depletion of mucin | 0.0 | 0.0 | 0.0 | 0.0 | 0.0 | 0.0 | 1.308 | 0.263 | 1.0 | 0.632 | 1.333 | 0.225 |
| Epithelial ulceration | 0.0 | 0.0 | 0.0 | 0.0 | 0.0 | 0.0 | 0.769 | 0.323 | 0.0 | 0.0 | 2.667 | 0.414 |
| Cytoarchitectural distortion | 0.0 | 0.0 | 0.0 | 0.0 | 0.0 | 0.0 | 1.154 | 0.249 | 1.4 | 0.510 | 2.00 | 0.213 |
| TOTAL | 0.0 | 0.0 | 0.0 | 0.0 | 0.231 | 0.122 | 5.923 | 1.118 | 3.8 | 1.772 | 9.833 | 0.976 |

**Table S3. Inflammatory cells on histological samples. NC: normal chow, HF: high-fat, HFHS: high-fat high-sucrose, DSS: dextran sulfate sodium.**

|  | NC DSS^-^  (N = 13) | | HF DSS^-^ (N = 5) | | HFHS DSS^-^  (N = 13) | | NC DSS^+^  (N = 13) | | HF DSS^+^ (N = 5) | | HFHS DSS^+^ (N = 12) | |
| --- | --- | --- | --- | --- | --- | --- | --- | --- | --- | --- | --- | --- |
| % | Mean | SEM | Mean | SEM | Mean | SEM | Mean | SEM | Mean | SEM | Mean | SEM |
| Lymphocytes | 0.0 | 0.0 | 0.0 | 0.0 | 44.286 | 11.518 | 33.571 | 7.377 | 47.000 | 19.339 | 46.250 | 4.605 |
| Neutrophils | 0.0 | 0.0 | 0.0 | 0.0 | 0.0 | 0.0 | 18.571 | 7.131 | 2.600 | 1.661 | 9.625 | 3.751 |
| Eosinophils | 0.0 | 0.0 | 0.0 | 0.0 | 0.0 | 0.0 | 10.000 | 3.934 | 0.400 | 0.400 | 9.125 | 1.856 |
| Plasmocytes | 0.0 | 0.0 | 0.0 | 0.0 | 11.429 | 5.1 | 22.143 | 3.912 | 5.000 | 2.236 | 24.375 | 3.464 |
| Histiocytes | 0.0 | 0.0 | 0.0 | 0.0 | 15.714 | 5.3 | 15.714 | 3.168 | 5.000 | 2.236 | 10.625 | 1.475 |

**Table S4.** **Functional annotations of the dysregulated genes in mice fed with normal chow or high-fat high-sucrose diet.** Top disease associations as obtained with the OpenTargets platform (genes = 164; p.value < 0.001; number of targets > 18).

| **Disease full name** | **Relevance (p-value)** | **Nb of associated targets** | **Therapeutic Area** | **Highest associated targets (max 10)** |
| --- | --- | --- | --- | --- |
| large intestine disease | 0.0000 | 93 | gastrointestinal disease | MSH6 BCL9 EIF3E HIF1A FRK EML4 EIF3H ARHGAP5 TCEA1 DNAJB1 |
| Gastroenteritis | 0.0001 | 43 | gastrointestinal disease | CUL2 MSH6 CAPN10 ZNF300 HIF1A EIF2S1 FLT3LG IGFBP2 DNAJB1 SLAMF7 |
| diarrheal disease | 0.0002 | 18 | gastrointestinal disease | FERMT1 DBNDD2 ESCO1 USP15 PKN2 DNAJC3 CACYBP HPN CLOCK HSPA5 |
| immune system disease | 0.0000 | 134 | immune system disease | MSH6 ARHGAP5 LRRFIP1 SLAMF7 RAP1A HIF1A MARK3 HSPA8 RGS1 BCL9 |
| autoimmune disease | 0.0000 | 80 | immune system disease | RGS1 CUL2 HSPA8 NAB1 FERMT1 PPP4R3B SMYD3 CAPN10 MSH6 ZNF300 |
| inflammatory bowel disease | 0.0002 | 47 | immune system disease gastrointestinal disease | CUL2 FERMT1 CAPN10 MSH6 ZNF300 RAP1A POLB DNAJC3 HIF1A USP15 |
| Colitis | 0.0005 | 37 | immune system disease gastrointestinal disease | CUL2 MSH6 CAPN10 ZNF300 HIF1A FLT3LG EIF2S1 DNAJB1 IGFBP2 SLAMF7 |
| immune deficiency disease | 0.0001 | 45 | immune system disease. genetic familial or congenital disease | MSH6 POLB FLT3LG FMR1 USP15 CLOCK CACYBP ESCO1 HSPA5 RAP1A |
| Fibrosis | 0.0002 | 37 | cell proliferation disorder | NAB1 HIF1A HSPA5 IGFBP2 FRK KPNA3 EIF2S1 FLT3LG MSH6 AMD1 |
| precancerous condition | 0.0000 | 31 | cell proliferation disorder | MSH6 ATP2B1 INTS12 HIF1A IGFBP2 BCL9 PRKX POLB USP15 HSPA5 |
| inherited digestive cancer-predisposing syndrome | 0.0000 | 21 | genetic. familial or congenital disease. cell proliferation disorder | MSH6 ATP2B1 INTS12 POLB HIF1A USP15 CAB39 TAOK1 ETF1 HSPA5 |
| endocrine system disease | 0.0000 | 128 | endocrine system disease | FMR1 AAAS MSH6 MARK3 HIF1A RAP1A EML4 BCL9 DNAJB1 EIF3E |
| diabetes mellitus | 0.0000 | 62 | pancreas disease. nutritional or metabolic disease | CAPN10 HPN BCL9 SPATA5 ZNF251 HSPA8 DNAJC3 CLOCK PPP4R3B FMR1 |
| type I diabetes mellitus | 0.0000 | 36 | pancreas disease. nutritional or metabolic disease. immune system disease. endocrine system disease | PPP4R3B CLOCK DNAJC3 HIF1A HSPH1 IGFBP2 HSPA1B HSPA5 NPB AMD1 |
| metabolic disease | 0.0000 | 117 | nutritional or metabolic disease | FMR1 UMPS MRPS22 PDP1 ALG2 PIGY CAPN10 FASTKD2 HPN BCL9 |
| glucose metabolism disease | 0.0000 | 61 | nutritional or metabolic disease | CAPN10 HPN BCL9 SPATA5 ZNF251 HSPA8 DNAJC3 CLOCK EIF2S1 PPP4R3B |
| Disorder of energy metabolism | 0.0000 | 41 | nutritional or metabolic disease. genetic. familial or congenital disease | MRPS22 PDP1 FASTKD2 HSPA5 ATP2B1 CLOCK UBE3A MARK3 EIF2S1 DNAJC3 |
| Disorder of lipid metabolism | 0.0001 | 39 | nutritional or metabolic disease. genetic. familial or congenital disease | PIGY N4BP2L2 HSPA5 POLB CLOCK TRA2B ERBIN UBE3A FERMT1 RNF139 |
| Mitochondrial disease | 0.0000 | 37 | nutritional or metabolic disease. genetic. familial or congenital disease | MRPS22 PDP1 FASTKD2 ATP2B1 HSPA5 UBE3A CLOCK RB1CC1 POLB MARK3 |
| carbohydrate metabolism disease | 0.0002 | 34 | nutritional or metabolic disease | PDP1 ALG2 PIGY EIF2S1 DNAJC3 HSPA5 MARK3 ATP2B1 HIF1A CLOCK |
| DNA repair deficiency | 0.0002 | 33 | nutritional or metabolic disease. genetic. familial or congenital disease | POLB ATP2B1 FLT3LG USP15 PKN2 DBNDD2 ESCO1 HSPA5 CHORDC1 RGS1 |
| Mitochondrial oxidative phosphorylation disorder | 0.0001 | 31 | nutritional or metabolic disease. genetic. familial or congenital disease | MRPS22 FASTKD2 HSPA5 ATP2B1 CLOCK UBE3A POLB RB1CC1 CHORDC1 MARK3 |
| disorder of glycosylation | 0.0010 | 26 | nutritional or metabolic disease | ALG2 PIGY ESCO1 DBNDD2 HSPA5 HPN UBE3A USP15 PKN2 CAPN10 |
| acquired metabolic disease | 0.0010 | 24 | nutritional or metabolic disease | FXR1 SLAMF7 CRY1 HIF1A HSPA5 ETF1 EIF2S1 POLB IGFBP2 HSPA8 |
| Mitochondrial disorder due to a defect in mitochondrial protein synthesis | 0.0010 | 18 | nutritional or metabolic disease. genetic. familial or congenital disease | MRPS22 ATP2B1 MARK3 ERBIN CHORDC1 RB1CC1 HIF1A GPBP1 CLOCK UBE3A |
| Disorder of protein N-glycosylation | 0.0010 | 18 | nutritional or metabolic disease. genetic. familial or congenital disease | ALG2 ESCO1 DBNDD2 USP15 PKN2 CACYBP HIF1A RNF139 ERBIN HPN |
| Abnormality of the immune system | 0.0000 | 75 | phenotype | AAAS CD200R1L PKN2 HIF1A HSPA5 EIF3E FLT3LG IGFBP2 DNAJC3 RGS1 |
| inflammation | 0.0000 | 56 | phenotype | HIF1A HSPA5 EIF3E FLT3LG DNAJC3 RGS1 CLOCK DNTTIP2 AMD1 EIF2S1 |
| Abnormality of metabolism/homeostasis | 0.0000 | 54 | phenotype | N4BP2L2 CLOCK MGA FAM210A EIF2S1 MARK3 DNAJC3 CYP2C9 HIF1A HSPA5 |
| Abnormality of the digestive system | 0.0000 | 47 | phenotype | PKN2 HSPA5 ESCO1 USP15 DBNDD2 HPN CACYBP CLOCK CYP2C9 EIF3M |
| Abnormality of the gastrointestinal tract | 0.0010 | 21 | phenotype | PKN2 UBA3 CYP2C9 ZNF512B HIF1A AMD1 FERMT1 HSPA5 IGFBP2 HPN |
| infectious disease | 0.0000 | 104 | infectious disease | TCEA1 ELOA PSMC6 TAF9 AAAS PSMD5 HIF1A ATP2B1 DNAJC3 TRA2B |
| viral disease | 0.0000 | 74 | infectious disease | TCEA1 ELOA AAAS TAF9 PSMD5 PSMC6 HIF1A DNAJC3 ATP2B1 HSPA5 |
| injury | 0.0000 | 67 | injury. poisoning or other complication | LURAP1L FAM210A HIF1A DNAJC3 CAPN10 FLT3LG IGFBP2 HSPA5 CYP2C9 AMD1 |
| bacterial disease | 0.0000 | 43 | infectious disease | HIF1A UMPS FLT3LG HSPA5 HSPA1B PDP1 CYP2C9 RB1CC1 EIF3M EFHB |
| complication | 0.0000 | 34 | injury. poisoning or other complication | CYP2C9 FAM210A HPN CAPN10 HIF1A AMD1 HSPA1B IGFBP2 HSPA5 PDP1 |
| digestive system infectious disease | 0.0001 | 32 | infectious disease. gastrointestinal disease | HSPA5 HIF1A AMD1 EIF2S1 FLT3LG ETF1 PKN2 NPB UBE3A SMYD3 |

**Table S5. Microbiota changes associated with high-fat high-sucrose diet.** The test numbers in the Table refers to the method section. NC: normal chow diet, HFHS: high-fat high-sucrose diet,

| **Alpha Diversity** | | **p-value of Index** | | | | | **p-value of Fold Change** | |
| --- | --- | --- | --- | --- | --- | --- | --- | --- |
|  |  | Chao | | Shannon | | | Chao | Shannon |
| **NC vs HFHS** | **B** | 0.0006 | | 0.0348 | | | NA | NA |
|  | **D0** | 0.0005 | | 0.0063 | | | 0.1051 | 0.1153 |
| **B vs D0** | **NC** | 0.0639 | | 0.2969 | | | 0.0018 | 0.1153 |
|  | **HFHS** | 0.0005 | | 0.0003 | | | 0.0002 | 0.0002 |
| **Beta Diversity** | | p.value | | | | | Variance | |
|  |  | Bray-Curtis | | | Jaccard | | Bray-Curtis | Jaccard |
| **NC vs HFHS** | **B** | 0.0001 | | | 0.0001 | | 52.5% | 42.2% |
|  | **D0** | 0.0001 | | | 0.0002 | | 47.5% | 49.0% |
| **B vs D0** | **NC** | 0.0242 | | | 0.0056 | | 14.2% | 13.0% |
|  | **HFHS** | 0.0001 | | | 0.0001 | | 71.1% | 25.8% |
| **Biomarkers** | | Otu036 | Otu044 | | | Otu052 | Otu146 | Otu194 |
| **Baseline** | **p-value test (1)** | 0.8404 | 0.3855 | | | 0.2918 | 0.2918 | 1.0000 |
|  | **p-value test (2)** | 1.0000 | 1.0000 | | | 0.8412 | 0.8412 | 1.0000 |
|  | **p-value test (3)** | 1.0000 | 1.0000 | | | 0.8592 | 0.8592 | 1.0000 |
|  | **p-value test (4)** | 0.7545 | 0.4103 | | | 0.3363 | 0.3363 | 1.0000 |
|  | **strength of association test (1)** | 0.3% | 4.9% | | | 7.7% | 7.7% | 0.0% |
|  | **strength of association test (2)** | 0.0% | 0.0% | | | 45.0% | 45.0% | 0.0% |
|  | **strength of association test (3)** | 0.0% | 0.0% | | | 25.0% | 25.0% | 0.0% |
|  | **strength of association test (4)** | 74.3% | 80.6% | | | 53.7% | 50.5% | 0.0% |
|  | **test (4) specificity** | 55.2% | 65.0% | | | 96.3% | 85.0% | 0.0% |
|  | **test (4) sensitivity** | 100.0% | 100.0% | | | 30.0% | 30.0% | 0.0% |
|  | **test (4) associated group** | NC | HFHS | | | NC | HFHS |  |
| **Day zero** | **p-value test (1)** | 0.0015 | 0.0018 | | | 0.0015 | 0.0014 | 0.0010 |
|  | **p-value test (2)** | 0.1021 | 0.1021 | | | 1.0000 | 0.2258 | 0.7482 |
|  | **p-value test (3)** | 0.1255 | 0.4157 | | | 0.0031 | 0.0258 | 0.0031 |
|  | **p-value test (4)** | 0.0008 | 0.0018 | | | 0.0028 | 0.0008 | 0.0008 |
|  | **strength of association test (1)** | 72.5% | 66.2% | | | 69.3% | 74.6% | 78.6% |
|  | **strength of association test (2)** | 62.5% | 61.3% | | | 0.0% | 49.6% | 22.9% |
|  | **strength of association test (3)** | 57.7% | 42.0% | | | 90.5% | 73.4% | 90.5% |
|  | **strength of association test (4)** | 98.0% | 94.1% | | | 94.9% | 98.3% | 99.1% |
|  | **test (4) specificity** | 96.0% | 88.5% | | | 100.0% | 96.5% | 98.2% |
|  | **test (4) sensitivity** | 100.0% | 100.0% | | | 90.0% | 100.0% | 100.0% |
|  | **test (4) associated group** | NC | NC | | | HFHS | HFHS | NC |
| **Fold Change** | **p-value test (1)** | 0.0132 | 0.0426 | | | 0.0150 | 0.0426 | 0.0091 |
|  | **strength of association test (1)** | 68.6% | 50.4% | | | 65.9% | 49.3% | 78.6% |
| **Taxonomy** | **Phylum** | Firmicutes | Firmicutes | | | Firmicutes | Firmicutes | Bacteroidetes |
|  | **Class** | Clostridia | Clostridia | | | Clostridia | Clostridia | Bacteroidia |
|  | **Order** | Clostridiales | Clostridiales | | | Clostridiales | Clostridiales | Bacteroidales |
|  | **Family** | unclassified | Lachnospiraceae | | | Lachnospiraceae | Lachnospiraceae | Porphyromonadaceae |
|  | **Genus** | unclassified | unclassified | | | unclassified | unclassified | Barnesiella |

**Table S6.** **Top Reactome pathways of dysregulated genes (over-representation test) in mice fed with high-fat high-sucrose diet, normal chow, or both.**

|  | Pathway identifier | Pathway name | #Entities found | #Entities total | Entities ratio | Entities pValue | Entities FDR | #Reactions found | #Reactions total | Reactions ratio |
| --- | --- | --- | --- | --- | --- | --- | --- | --- | --- | --- |
| Cluster 1 | R-HSA-381038 | XBP1(S) activates chaperone genes | 2 | 95 | 0.007 | 0.00 | 0.082 | 1 | 47 | 0.004 |
|  | R-HSA-8986944 | Transcriptional Regulation by MECP2 | 2 | 100 | 0.007 | 0.01 | 0.082 | 2 | 77 | 0.006 |
|  | R-HSA-381070 | IRE1alpha activates chaperones | 2 | 101 | 0.007 | 0.01 | 0.082 | 1 | 53 | 0.004 |
|  | R-HSA-2672351 | Stimuli-sensing channels | 2 | 120 | 0.008 | 0.01 | 0.082 | 2 | 26 | 0.002 |
|  | R-HSA-5263617 | Metabolism of ingested MeSeO2H into MeSeH | 1 | 9 | 0.001 | 0.01 | 0.082 | 2 | 2 | 0.000 |
|  | R-HSA-3371599 | Defective HLCS causes multiple carboxylase deficiency | 1 | 10 | 0.001 | 0.01 | 0.082 | 1 | 4 | 0.000 |
|  | R-HSA-5336415 | Uptake and function of diphtheria toxin | 1 | 10 | 0.001 | 0.01 | 0.082 | 1 | 5 | 0.000 |
|  | R-HSA-381119 | Unfolded Protein Response (UPR) | 2 | 155 | 0.011 | 0.01 | 0.082 | 1 | 94 | 0.007 |
|  | R-HSA-3323169 | Defects in biotin (Btn) metabolism | 1 | 12 | 0.001 | 0.01 | 0.082 | 1 | 6 | 0.000 |
| Cluster 2 | R-HSA-8939243 | RUNX1 interacts with co-factors whose precise effect on RUNX1 targets is not known | 3 | 39 | 0.003 | 0.00 | 0.023 | 2 | 5 | 0.000 |
|  | R-HSA-8943724 | Regulation of PTEN gene transcription | 3 | 70 | 0.005 | 0.00 | 0.043 | 3 | 15 | 0.001 |
|  | R-HSA-4655427 | SUMOylation of DNA methylation proteins | 2 | 16 | 0.001 | 0.00 | 0.043 | 1 | 3 | 0.000 |
|  | R-HSA-3899300 | SUMOylation of transcription cofactors | 2 | 44 | 0.003 | 0.01 | 0.159 | 4 | 27 | 0.002 |
|  | R-HSA-4570464 | SUMOylation of RNA binding proteins | 2 | 51 | 0.004 | 0.01 | 0.159 | 1 | 4 | 0.000 |
|  | R-HSA-6807070 | PTEN Regulation | 3 | 171 | 0.012 | 0.01 | 0.159 | 3 | 56 | 0.004 |
|  | R-HSA-4551638 | SUMOylation of chromatin organization proteins | 2 | 62 | 0.004 | 0.01 | 0.159 | 2 | 15 | 0.001 |
|  | R-HSA-9636569 | Suppression of autophagy | 1 | 7 | 0.000 | 0.02 | 0.159 | 1 | 2 | 0.000 |
| Cluster 3 | R-HSA-69560 | Transcriptional activation of p53 responsive genes | 3 | 6 | 0,000 | 0,00 | 0,000 | 4 | 5 | 0,000 |
|  | R-HSA-69895 | Transcriptional activation of cell cycle inhibitor p21 | 3 | 6 | 0,000 | 0,00 | 0,000 | 4 | 5 | 0,000 |
|  | R-HSA-6804116 | TP53 Regulates Transcription of Genes Involved in G1 Cell Cycle Arrest | 3 | 20 | 0,001 | 0,00 | 0,007 | 6 | 17 | 0,001 |
|  | R-HSA-69563 | p53-Dependent G1 DNA Damage Response | 4 | 70 | 0,005 | 0,00 | 0,008 | 7 | 22 | 0,002 |
|  | R-HSA-69580 | p53-Dependent G1/S DNA damage checkpoint | 4 | 70 | 0,005 | 0,00 | 0,008 | 7 | 22 | 0,002 |
|  | R-HSA-9617828 | FOXO-mediated transcription of cell cycle genes | 3 | 27 | 0,002 | 0,00 | 0,008 | 5 | 22 | 0,002 |
|  | R-HSA-69615 | G1/S DNA Damage Checkpoints | 4 | 72 | 0,005 | 0,00 | 0,008 | 8 | 26 | 0,002 |
|  | R-HSA-8866911 | TFAP2 (AP-2) family regulates transcription of cell cycle factors | 2 | 6 | 0,000 | 0,00 | 0,010 | 3 | 4 | 0,000 |
|  | R-HSA-5635851 | GLI proteins bind promoters of Hh responsive genes to promote transcription | 2 | 8 | 0,001 | 0,00 | 0,015 | 4 | 4 | 0,000 |
|  | R-HSA-8941855 | RUNX3 regulates CDKN1A transcription | 2 | 8 | 0,001 | 0,00 | 0,015 | 3 | 6 | 0,000 |
|  | R-HSA-5610787 | Hedgehog 'off' state | 4 | 124 | 0,009 | 0,00 | 0,039 | 12 | 32 | 0,003 |
|  | R-HSA-6791312 | TP53 Regulates Transcription of Cell Cycle Genes | 3 | 65 | 0,004 | 0,00 | 0,053 | 6 | 42 | 0,003 |
|  | R-HSA-201722 | Formation of the beta-catenin:TCF transactivating complex | 3 | 67 | 0,005 | 0,00 | 0,054 | 4 | 13 | 0,001 |
|  | R-HSA-2559586 | DNA Damage/Telomere Stress Induced Senescence | 3 | 71 | 0,005 | 0,00 | 0,058 | 7 | 18 | 0,001 |
|  | R-HSA-5358351 | Signaling by Hedgehog | 4 | 168 | 0,012 | 0,00 | 0,084 | 27 | 82 | 0,006 |
|  | R-HSA-5632684 | Hedgehog 'on' state | 3 | 92 | 0,006 | 0,01 | 0,105 | 15 | 37 | 0,003 |
|  | R-HSA-8939236 | RUNX1 regulates transcription of genes involved in differentiation of HSCs | 3 | 106 | 0,007 | 0,01 | 0,136 | 7 | 15 | 0,001 |
|  | R-HSA-210745 | Regulation of gene expression in beta cells | 2 | 35 | 0,002 | 0,01 | 0,136 | 1 | 12 | 0,001 |
|  | R-HSA-162710 | Synthesis of glycosylphosphatidylinositol (GPI) | 2 | 36 | 0,002 | 0,01 | 0,136 | 1 | 11 | 0,001 |
|  | R-HSA-9614085 | FOXO-mediated transcription | 3 | 110 | 0,008 | 0,01 | 0,136 | 5 | 85 | 0,007 |
|  | R-HSA-201681 | TCF dependent signaling in response to WNT | 4 | 216 | 0,015 | 0,01 | 0,137 | 9 | 71 | 0,006 |
|  | R-HSA-8878159 | Transcriptional regulation by RUNX3 | 3 | 118 | 0,008 | 0,01 | 0,154 | 4 | 47 | 0,004 |
|  | R-HSA-1368108 | BMAL1:CLOCK,NPAS2 activates circadian gene expression | 2 | 42 | 0,003 | 0,01 | 0,157 | 2 | 20 | 0,002 |
|  | R-HSA-212300 | PRC2 methylates histones and DNA | 2 | 44 | 0,003 | 0,01 | 0,159 | 4 | 4 | 0,000 |
|  | R-HSA-3769402 | Deactivation of the beta-catenin transactivating complex | 2 | 44 | 0,003 | 0,01 | 0,159 | 1 | 14 | 0,001 |
| Cluster 4 | R-HSA-3371568 | Attenuation phase | 11 | 47 | 0,003 | 0,00 | 0,00 | 3 | 5 | 0,00 |
|  | R-HSA-3371453 | Regulation of HSF1-mediated heat shock response | 15 | 113 | 0,008 | 0,00 | 0,00 | 8 | 14 | 0,00 |
|  | R-HSA-3371571 | HSF1-dependent transactivation | 11 | 59 | 0,004 | 0,00 | 0,00 | 4 | 8 | 0,00 |
|  | R-HSA-8953854 | Metabolism of RNA | 45 | 782 | 0,054 | 0,00 | 0,00 | 61 | 187 | 0,01 |
|  | R-HSA-3371556 | Cellular response to heat stress | 15 | 135 | 0,009 | 0,00 | 0,00 | 13 | 29 | 0,00 |
|  | R-HSA-3371511 | HSF1 activation | 8 | 43 | 0,003 | 0,00 | 0,01 | 1 | 7 | 0,00 |
|  | R-HSA-8951664 | Neddylation | 18 | 241 | 0,017 | 0,00 | 0,05 | 24 | 31 | 0,00 |
|  | R-HSA-8948751 | Regulation of PTEN stability and activity | 9 | 74 | 0,005 | 0,00 | 0,05 | 6 | 13 | 0,00 |
|  | R-HSA-2262752 | Cellular responses to stress | 36 | 690 | 0,048 | 0,00 | 0,07 | 67 | 227 | 0,02 |
|  | R-HSA-186763 | Downstream signal transduction | 6 | 37 | 0,003 | 0,00 | 0,09 | 15 | 16 | 0,00 |
|  | R-HSA-8953897 | Cellular responses to external stimuli | 36 | 708 | 0,049 | 0,00 | 0,09 | 67 | 258 | 0,02 |
|  | R-HSA-68867 | Assembly of the pre-replicative complex | 8 | 68 | 0,005 | 0,00 | 0,09 | 7 | 12 | 0,00 |
|  | R-HSA-983168 | Antigen processing: Ubiquitination & Proteasome degradation | 20 | 315 | 0,022 | 0,00 | 0,09 | 7 | 9 | 0,00 |
|  | R-HSA-68882 | Mitotic Anaphase | 17 | 248 | 0,017 | 0,00 | 0,09 | 16 | 32 | 0,00 |
|  | R-HSA-2555396 | Mitotic Metaphase and Anaphase | 17 | 249 | 0,017 | 0,00 | 0,09 | 16 | 33 | 0,00 |
|  | R-HSA-69002 | DNA Replication Pre-Initiation | 9 | 88 | 0,006 | 0,00 | 0,09 | 15 | 21 | 0,00 |
|  | R-HSA-450408 | AUF1 (hnRNP D0) binds and destabilizes mRNA | 7 | 56 | 0,004 | 0,00 | 0,09 | 3 | 4 | 0,00 |
|  | R-HSA-4641257 | Degradation of AXIN | 7 | 57 | 0,004 | 0,00 | 0,10 | 6 | 8 | 0,00 |
|  | R-HSA-1632852 | Macroautophagy | 12 | 150 | 0,010 | 0,00 | 0,10 | 41 | 87 | 0,01 |
|  | R-HSA-6807070 | PTEN Regulation | 13 | 171 | 0,012 | 0,00 | 0,10 | 17 | 56 | 0,00 |
|  | R-HSA-68827 | CDT1 association with the CDC6:ORC:origin complex | 7 | 59 | 0,004 | 0,00 | 0,10 | 3 | 3 | 0,00 |
|  | R-HSA-112382 | Formation of RNA Pol II elongation complex | 7 | 63 | 0,004 | 0,00 | 0,14 | 2 | 2 | 0,00 |
|  | R-HSA-72702 | Ribosomal scanning and start codon recognition | 7 | 64 | 0,004 | 0,00 | 0,14 | 2 | 2 | 0,00 |
|  | R-HSA-72163 | mRNA Splicing - Major Pathway | 13 | 185 | 0,013 | 0,00 | 0,15 | 9 | 9 | 0,00 |
|  | R-HSA-75955 | RNA Polymerase II Transcription Elongation | 7 | 66 | 0,005 | 0,00 | 0,15 | 8 | 8 | 0,00 |
|  | R-HSA-9612973 | Autophagy | 12 | 166 | 0,011 | 0,00 | 0,17 | 52 | 108 | 0,01 |
|  | R-HSA-8873719 | RAB geranylgeranylation | 7 | 68 | 0,005 | 0,00 | 0,17 | 3 | 5 | 0,00 |
|  | R-HSA-674695 | RNA Polymerase II Pre-transcription Events | 8 | 88 | 0,006 | 0,00 | 0,17 | 12 | 17 | 0,00 |
|  | R-HSA-2467813 | Separation of Sister Chromatids | 13 | 194 | 0,013 | 0,01 | 0,17 | 4 | 8 | 0,00 |
|  | R-HSA-5658442 | Regulation of RAS by GAPs | 7 | 71 | 0,005 | 0,01 | 0,17 | 4 | 5 | 0,00 |
|  | R-HSA-72737 | Cap-dependent Translation Initiation | 10 | 130 | 0,009 | 0,01 | 0,17 | 14 | 18 | 0,00 |
|  | R-HSA-72613 | Eukaryotic Translation Initiation | 10 | 130 | 0,009 | 0,01 | 0,17 | 16 | 21 | 0,00 |
|  | R-HSA-72695 | Formation of the ternary complex, and subsequently, the 43S complex | 6 | 54 | 0,004 | 0,01 | 0,17 | 3 | 3 | 0,00 |
|  | R-HSA-72172 | mRNA Splicing | 13 | 196 | 0,014 | 0,01 | 0,17 | 14 | 14 | 0,00 |
|  | R-HSA-1234176 | Oxygen-dependent proline hydroxylation of Hypoxia-inducible Factor Alpha | 7 | 72 | 0,005 | 0,01 | 0,17 | 10 | 12 | 0,00 |
|  | R-HSA-68949 | Orc1 removal from chromatin | 7 | 73 | 0,005 | 0,01 | 0,17 | 2 | 4 | 0,00 |
|  | R-HSA-6807505 | RNA polymerase II transcribes snRNA genes | 8 | 94 | 0,006 | 0,01 | 0,19 | 10 | 11 | 0,00 |
|  | R-HSA-5689880 | Ub-specific processing proteases | 13 | 206 | 0,014 | 0,01 | 0,22 | 8 | 40 | 0,00 |
|  | R-HSA-69620 | Cell Cycle Checkpoints | 16 | 279 | 0,019 | 0,01 | 0,22 | 16 | 56 | 0,00 |
|  | R-HSA-72706 | GTP hydrolysis and joining of the 60S ribosomal subunit | 9 | 120 | 0,008 | 0,01 | 0,22 | 3 | 3 | 0,00 |
|  | R-HSA-5607764 | CLEC7A (Dectin-1) signaling | 9 | 120 | 0,008 | 0,01 | 0,22 | 9 | 45 | 0,00 |
|  | R-HSA-9639288 | Amino acids regulate mTORC1 | 6 | 61 | 0,004 | 0,01 | 0,22 | 9 | 12 | 0,00 |
|  | R-HSA-5676590 | NIK-->noncanonical NF-kB signaling | 6 | 61 | 0,004 | 0,01 | 0,22 | 2 | 9 | 0,00 |
|  | R-HSA-983169 | Class I MHC mediated antigen processing & presentation | 23 | 465 | 0,032 | 0,01 | 0,22 | 19 | 48 | 0,00 |
|  | R-HSA-72649 | Translation initiation complex formation | 6 | 62 | 0,004 | 0,01 | 0,22 | 2 | 2 | 0,00 |
|  | R-HSA-9607240 | FLT3 Signaling | 17 | 311 | 0,021 | 0,01 | 0,22 | 29 | 61 | 0,00 |
|  | R-HSA-162906 | HIV Infection | 15 | 262 | 0,018 | 0,01 | 0,22 | 51 | 158 | 0,01 |
|  | R-HSA-9006925 | Intracellular signaling by second messengers | 19 | 363 | 0,025 | 0,01 | 0,22 | 21 | 114 | 0,01 |
|  | R-HSA-5674135 | MAP2K and MAPK activation | 6 | 63 | 0,004 | 0,01 | 0,22 | 6 | 8 | 0,00 |
|  | R-HSA-5688426 | Deubiquitination | 16 | 288 | 0,020 | 0,01 | 0,22 | 16 | 77 | 0,01 |
|  | R-HSA-73772 | RNA Polymerase I Promoter Escape | 6 | 64 | 0,004 | 0,01 | 0,22 | 2 | 2 | 0,00 |
|  | R-HSA-5673001 | RAF/MAP kinase cascade | 16 | 290 | 0,020 | 0,01 | 0,22 | 25 | 39 | 0,00 |
|  | R-HSA-1257604 | PIP3 activates AKT signaling | 17 | 316 | 0,022 | 0,01 | 0,22 | 19 | 86 | 0,01 |
|  | R-HSA-1234174 | Cellular response to hypoxia | 7 | 85 | 0,006 | 0,01 | 0,22 | 18 | 20 | 0,00 |
|  | R-HSA-4608870 | Asymmetric localization of PCP proteins | 6 | 66 | 0,005 | 0,01 | 0,22 | 4 | 7 | 0,00 |
|  | R-HSA-72662 | Activation of the mRNA upon binding of the cap-binding complex and eIFs, and subsequent binding to 43S | 6 | 66 | 0,005 | 0,01 | 0,22 | 2 | 6 | 0,00 |
|  | R-HSA-5607761 | Dectin-1 mediated noncanonical NF-kB signaling | 6 | 66 | 0,005 | 0,01 | 0,22 | 2 | 9 | 0,00 |
|  | R-HSA-453279 | Mitotic G1 phase and G1/S transition | 11 | 173 | 0,012 | 0,01 | 0,22 | 16 | 98 | 0,01 |
|  | R-HSA-186797 | Signaling by PDGF | 7 | 86 | 0,006 | 0,01 | 0,22 | 24 | 31 | 0,00 |
|  | R-HSA-5684996 | MAPK1/MAPK3 signaling | 16 | 297 | 0,020 | 0,01 | 0,22 | 25 | 46 | 0,00 |
|  | R-HSA-167200 | Formation of HIV-1 elongation complex containing HIV-1 Tat | 5 | 49 | 0,003 | 0,01 | 0,22 | 4 | 5 | 0,00 |
| Cluster 5 | R-HSA-73779 | RNA Polymerase II Transcription Pre-Initiation And Promoter Opening | 4 | 47 | 0,003 | 0,00 | 0,01 | 5 | 5 | 0,00 |
|  | R-HSA-167162 | RNA Polymerase II HIV Promoter Escape | 4 | 52 | 0,004 | 0,00 | 0,01 | 9 | 9 | 0,00 |
|  | R-HSA-167161 | HIV Transcription Initiation | 4 | 52 | 0,004 | 0,00 | 0,01 | 4 | 4 | 0,00 |
|  | R-HSA-75953 | RNA Polymerase II Transcription Initiation | 4 | 52 | 0,004 | 0,00 | 0,01 | 3 | 3 | 0,00 |
|  | R-HSA-73776 | RNA Polymerase II Promoter Escape | 4 | 53 | 0,004 | 0,00 | 0,01 | 7 | 7 | 0,00 |
|  | R-HSA-76042 | RNA Polymerase II Transcription Initiation And Promoter Clearance | 4 | 55 | 0,004 | 0,00 | 0,01 | 11 | 11 | 0,00 |
|  | R-HSA-8953854 | Metabolism of RNA | 12 | 782 | 0,054 | 0,00 | 0,01 | 52 | 187 | 0,01 |
|  | R-HSA-6803529 | FGFR2 alternative splicing | 3 | 28 | 0,002 | 0,00 | 0,01 | 4 | 4 | 0,00 |
|  | R-HSA-167172 | Transcription of the HIV genome | 4 | 81 | 0,006 | 0,00 | 0,01 | 46 | 47 | 0,00 |
| C6 | R-HSA-70263 | Gluconeogenesis | 2 | 34 | 0,003 | 0,00 | 0,049 | 2 | 25 | 0,002 |
|  | R-HSA-3274531 | Glycogen storage disease type Ia (G6PC) | 1 | 1 | 0,000 | 0,00 | 0,049 | 1 | 1 | 0,000 |
|  | R-HSA-70326 | Glucose metabolism | 2 | 98 | 0,009 | 0,01 | 0,168 | 2 | 49 | 0,004 |

**Table S7. Functional annotations of the dysregulated genes in HFHS+NC/NC/HFHS mice.** Top disease associations as obtained with the OpenTargets platform (genes = 75; *p*.value < 0.090; number of targets > 10). Top differential NC *versus* HFHS+NC genes from clusters 2 and 5.

| **Disease full name** | **Relevance (p-value)** | **Nb of associated targets** | **Therapeutic Area** | **Highest associated targets (max 10)** |
| --- | --- | --- | --- | --- |
| immune system disease | 0.0000 | 61 | immune system disease | TAZ PTCH1 NDUFAF4 GATAD2B MED4 POLR2A OR2L13 PHC2 DUPD1 NUFIP1 |
| autoimmune disease | 0.0000 | 50 | immune system disease | NDUFAF4 PTCH1 MED4 DUPD1 IQCH AFMID PKN3 LCE3C C1orf174 SIAH1 |
| immune system cancer | 0.0000 | 34 | immune system disease, cell proliferation disorder | PTCH1 GATAD2B MTRR OCIAD1 C1orf174 TBCC WDR43 EXOSC3 PLCD1 BICRA |
| inflammatory bowel disease | 0.0001 | 27 | immune system disease, gastrointestinal disease | PTCH1 MED4 DUPD1 IQCH LCE3C SIAH1 TTLL10 PHC2 WDR43 EXOC3 |
| colitis | 0.0002 | 22 | immune system disease, gastrointestinal disease | PTCH1 IQCH LCE3C SIAH1 PHC2 TTLL10 EXOC3 WDR43 UTP4 EXOSC3 |
| systemic autoimmune disease | 0.0004 | 20 | immune system disease | NDUFAF4 IQCH SCAMP2 LCE3C DUSP16 TBCC TAZ RRP8 RUVBL1 POLR2C |
| ulcerative colitis | 0.0200 | 20 | immune system disease, gastrointestinal disease | PTCH1 IQCH LCE3C SIAH1 TTLL10 EXOC3 WDR43 UTP4 EXOSC3 UBAC1 |
| Crohn's disease | 0.0900 | 15 | immune system disease, gastrointestinal disease | IQCH MED4 LCE3C TTLL10 MTRR MCRIP2 CCDC86 UBAC1 OCIAD1 TIMM10 |
| gastrointestinal disease | 0.0000 | 60 | gastrointestinal disease | AAAS PTCH1 DUSP16 NDUFAF4 TIMM23 MED4 POLR2A TAZ PHC2 SRSF6 |
| digestive system cancer | 0.0000 | 49 | gastrointestinal disease, cell proliferation disorder | PTCH1 DUSP16 NDUFAF4 TIMM23 TAZ SRSF6 IPO13 POLR2A GATAD2B UTP4 |
| colorectal cancer | 0.0000 | 41 | gastrointestinal disease, cell proliferation disorder | PTCH1 DUSP16 NDUFAF4 ELP1 SIAH1 MTRR PLCD1 RUVBL1 SRSF6 POLR2A |
| gastroenteritis | 0.0001 | 25 | gastrointestinal disease | PTCH1 IQCH LCE3C POLR2A SIAH1 PHC2 TTLL10 EXOC3 WDR43 UTP4 |
| nutritional or metabolic disease | 0.0000 | 54 | nutritional or metabolic disease | PIGA TAZ MTRR NDUFAF4 SENP2 IP6K2 PTCH1 SMN1 PHC2 SOX5 |
| diabetes mellitus | 0.0000 | 37 | pancreas disease, nutritional or metabolic disease | NDUFAF4 SENP2 AFMID PKN3 IQCH SMN1 PPIL3 PBX4 HNRNPF CRY1 |
| glucose metabolism disease | 0.0000 | 36 | nutritional or metabolic disease | NDUFAF4 SENP2 AFMID PKN3 IQCH SMN1 PPIL3 PBX4 HNRNPF CRY1 |
| type I diabetes mellitus | 0.0000 | 25 | pancreas disease, nutritional or metabolic disease, immune system disease, endocrine system disease | NDUFAF4 AFMID PKN3 IQCH MTRR TAZ RPP21 EXOSC3 HNRNPF SENP2 |
| nutritional disorder | 0.0030 | 23 | nutritional or metabolic disease | MTRR NDUFAF4 PTCH1 SOX5 PPIL3 SMN1 TAZ SIAH1 IP6K2 PLCD1 |
| overnutrition | 0.0090 | 20 | nutritional or metabolic disease | NDUFAF4 PTCH1 SOX5 PPIL3 TAZ SIAH1 MTRR PLCD1 CRY1 TBCC |
| Disorder of lipid metabolism | 0.0009 | 20 | nutritional or metabolic disease, genetic, familial or congenital disease | PIGA TAZ NDUFAF4 SOX5 PPIL3 SENP2 NSUN5 SMN1 PTCH1 PLCD1 |
| type II diabetes mellitus | 0.0020 | 19 | pancreas disease, nutritional or metabolic disease | NDUFAF4 SENP2 PBX4 MTRR PDF GTF3C4 HNRNPF UBAC1 OR2L13 DUPD1 |
| DNA repair deficiency | 0.0020 | 17 | nutritional or metabolic disease, genetic, familial or congenital disease | NDUFAF4 PTCH1 IP6K2 PHC2 PPIL3 RUVBL1 EXOSC3 MTRR TIMM23 SENP2 |
| Disorder of energy metabolism | 0.0200 | 15 | nutritional or metabolic disease, genetic, familial or congenital disease | TAZ NDUFAF4 SMN1 SOX5 AFMID SENP2 PPIL3 ELP1 OCIAD1 NSUN5 |
| carbohydrate metabolism disease | 0.0200 | 14 | nutritional or metabolic disease | PIGA PHC2 SOX5 ELP1 PPIL3 SMN1 SENP2 AFMID NSUN5 SCAMP2 |
| Mitochondrial disease | 0.0200 | 14 | nutritional or metabolic disease, genetic, familial or congenital disease | TAZ NDUFAF4 SMN1 SOX5 AFMID SENP2 ELP1 NSUN5 OCIAD1 TBCC |
| disorder of glycosylation | 0.0200 | 12 | nutritional or metabolic disease | PIGA PHC2 SOX5 SMN1 ELP1 PPIL3 NSUN5 SENP2 PTCH1 SCAMP2 |
| Lysosomal disease | 0.0050 | 12 | nutritional or metabolic disease, genetic, familial or congenital disease | SOX5 ELP1 NSUN5 SENP2 SMN1 SCAMP2 EXOSC3 CRY1 PTCH1 TBCC |
| Mitochondrial oxidative phosphorylation disorder | 0.0500 | 11 | nutritional or metabolic disease, genetic, familial or congenital disease | NDUFAF4 SMN1 SOX5 AFMID ELP1 SENP2 NSUN5 TBCC MTRR EXOSC3 |
| cancer | 0.0000 | 69 | cell proliferation disorder | PTCH1 DUSP16 NDUFAF4 POLR2A CBX4 GATAD2B RUVBL1 POLR2C PHC2 PLCD1 |
| carcinoma | 0.0000 | 63 | cell proliferation disorder | PTCH1 DUSP16 NDUFAF4 POLR2A CBX4 RUVBL1 POLR2C GATAD2B PHC2 HNRNPF |
| fibrosis | 0.0080 | 17 | cell proliferation disorder | PBX4 UTP4 SENP2 EXOSC3 PTCH1 HNRNPF TBCC MTRR PLCD1 LCE3C |
| infectious disease | 0.0000 | 44 | infectious disease | POLR2A DUSP16 AAAS POLR2D TAF12 POLR2C NDUFAF4 EXOSC3 SIAH1 NSUN5 |
| viral disease | 0.0002 | 29 | infectious disease | POLR2A POLR2D AAAS NDUFAF4 POLR2C TAF12 TBCC EXOSC3 SIAH1 SENP2 |
| bacterial disease | 0.0100 | 17 | infectious disease | DUSP16 MTRR EXOSC3 RRP8 PDF TBCC EXOC3 SMN1 PLCD1 TIMM23 |
| digestive system infectious disease | 0.0300 | 12 | infectious disease, gastrointestinal disease | NDUFAF4 EXOSC3 SIAH1 TBCC SMN1 MTRR RPP21 CRY1 RRP8 DUPD1 |
| injury | 0.0000 | 35 | injury, poisoning or other complication | NDUFAF4 OCIAD1 HNRNPF SIAH1 CRY1 EXOSC3 SOX5 PTCH1 PLCD1 AFMID |
| complication | 0.0200 | 13 | injury, poisoning or other complication | MTRR TBCC EXOSC3 PHC2 ELP1 SMN1 PIGA ATXN7L1 DUPD1 BICRA |
| Abnormality of the immune system | 0.0001 | 32 | phenotype | AAAS MED4 NDUFAF4 PKN3 IQCH EXOSC3 SNAPC1 MTRR TBCC DUSP16 |
| Abnormality of metabolism/homeostasis | 0.0000 | 31 | phenotype | MTRR NDUFAF4 PHC2 EXOSC3 CRY1 TAZ EXOC3 PIGA PTCH1 AFMID |
| Abnormality of the digestive system | 0.0020 | 21 | phenotype | NDUFAF4 PPIL3 SNAPC1 MTRR PTCH1 EXOSC3 TBCC SIAH1 RUVBL1 DUSP16 |
| inflammation | 0.0100 | 20 | phenotype | EXOSC3 NDUFAF4 MTRR DUSP16 PTCH1 PPIL3 TBCC CRY1 PLCD1 SOX5 |
| Increased inflammatory response | 0.0020 | 17 | phenotype | NDUFAF4 PKN3 IQCH SNAPC1 ZMYND19 LCE3C TIMM23 TBCC EXOSC3 RUVBL1 |
| Abnormality of body weight | 0.0080 | 11 | phenotype | NDUFAF4 EXOSC3 PLCD1 CBX4 PTCH1 TBCC MTRR ELP1 RPS19BP1 CRY1 |

**Table S8.** **Microbiota changes associated with high-fat high-sucrose diet switching to normal chow diet.** The test numbers in the Table refer to the method section. NC: normal chow diet, HFHS: high-fat high-sucrose diet, D0: day zero.

| **Alpha Diversity** | | | **p-value of Index** | | | | **p-value of Fold Change** | | | |
| --- | --- | --- | --- | --- | --- | --- | --- | --- | --- | --- |
|  |  |  | **Chao** | **Shannon** | | | **Chao** | | | **Shannon** |
| **NC vs. HFHS+NC** | | **B** | 0.0290 | 0.3374 | | | NA | | NA | |
|  |  | **D0** | 0.0010 | 0.0083 | | | 0.0786 | | 0.1729 | |
| **NC vs. HFHS** | | **B** | 0.0253 | 0.4913 | | | NA | | NA | |
|  |  | **D0** | 0.0000 | 0.0001 | | | 0.4429 | | 0.4200 | |
| **HFHS+NC vs. HFHS** | | **B** | 0.8534 | 0.6102 | | | NA | | NA | |
|  |  | **D0** | 0.0000 | 0.0001 | | | 0.0025 | | 0.0009 | |
| **B vs. D0** | | **NC** | 0.4670 | 0.7959 | | | 0.4429 | | 1.0000 | |
|  |  | **HFHS+NC** | 0.0134 | 0.0001 | | | 0.0295 | | 0.0043 | |
|  |  | **HFHS** | 0.0028 | 0.3374 | | | 0.0043 | | 0.1729 | |
| **Beta Diversity** | | | **p.value** | | | | **Variance** | | | |
|  |  |  | **Bray-Curtis** | | **Jaccard** | | **Bray-Curtis** | | | **Jaccard** |
| **NC vs. HFHS+NC** | | **B** | 0.0001 | 0.0001 | | | 30.8% | 38.4% | | |
|  |  | **D0** | 0.0004 | 0.0001 | | | 37.9% | 29.2% | | |
| **NC vs. HFHS** | | **B** | 0.0003 | 0.0002 | | | 30.4% | 36.0% | | |
|  |  | **D0** | 0.0001 | 0.0002 | | | 69.4% | 53.6% | | |
| **HFHS+NC vs. HFHS** | | **B** | 0.3427 | 0.1889 | | | 5.4% | 6.2% | | |
|  |  | **D0** | 0.0001 | 0.0001 | | | 57.7% | 47.3% | | |
| **B vs. D0** | | **NC** | 0.0001 | 0.0002 | | | 26.8% | 21.8% | | |
|  |  | **HFHS+NC** | 0.0001 | 0.0001 | | | 54.9% | 44.5% | | |
|  |  | **HFHS** | 0.0154 | 0.0001 | | | 17.9% | 22.3% | | |
| **Biomarkers** | | **Otu036** | **Otu044** | **Otu052** | | **Otu146** | **Otu194** | | | |
| **Baseline** | **p-value test (1)** | 0.2943 | 0.8073 | 0.0497 | | 0.4579 | 0.0005 | | | |
|  | **p-value test (2)** | 0.8863 | 1.0000 | 1.0000 | | 1.0000 | 1.0000 | | | |
|  | **p-value test (3)** | 0.5009 | 0.5077 | 0.0446 | | 0.4928 | 0.0003 | | | |
|  | **p-value test (4)** | 0.0764 | 1.0000 | 0.8742 | | 0.6875 | 0.0011 | | | |
|  | **strength of association test (1)** | 11.0% | 2.0% | 26.2% | | 7.4% | 68.2% | | | |
|  | **strength of association test (2)** | 8.5% | 2.9% | 0.2% | | 15.0% | 0.0% | | | |
|  | **strength of association test (3)** | 27.2% | 26.3% | 53.5% | | 27.7% | 85.3% | | | |
|  | **strength of association test (4)** | 75.0% | 59.6% | 45.8% | | 38.4% | 89.4% | | | |
|  | **test (4) specificity** | 56.2% | 35.5% | 35.0% | | 73.9% | 100.0% | | | |
|  | **test (4) sensitivity** | 100.0% | 100.0% | 60.0% | | 20.0% | 80.0% | | | |
|  | **test (4) associated group** | NC | HFHS+NC | HFHS+NC | | HFHS+NC | NC | | | |
| **Day zero** | **p-value test (1)** | 0.0001 | 0.0003 | 0.0001 | | 0.1086 | 0.0005 | | | |
|  | **p-value test (2)** | 0.0205 | 0.0375 | 1.0000 | | 0.2456 | 0.8265 | | | |
|  | **p-value test (3)** | 0.0002 | 0.0089 | 0.0000 | | 0.1221 | 0.0001 | | | |
|  | **p-value test (4)** | 0.0006 | 0.0317 | 0.0006 | | 0.1701 | 0.0039 | | | |
|  | **strength of association test (1)** | 82.5% | 66.8% | 91.6% | | 17.7% | 62.1% | | | |
|  | **strength of association test (2)** | 66.8% | 49.5% | 0.0% | | 51.0% | 4.6% | | | |
|  | **strength of association test (3)** | 85.3% | 63.2% | 100.0% | | 42.6% | 88.2% | | | |
|  | **strength of association test (4)** | 89.3% | 75.1% | 100.0% | | 59.6% | 78.0% | | | |
|  | **test (4) specificity** | 79.8% | 56.4% | 100.0% | | 88.9% | 60.9% | | | |
|  | **test (4) sensitivity** | 100.0% | 100.0% | 100.0% | | 40.0% | 100.0% | | | |
|  | **test (4) associated group** | NC | NC | HFHS | | HFHS | NC | | | |
| **Fold Change** | **p-value test (1)** | 0.0142 | 0.0031 | 0.0008 | | 0.3545 | 0.0158 | | | |
|  | **strength of association test (1)** | 39.4% | 53.7% | 69.8% | | 9.5% | 37.6% | | | |
| **Taxonomy** | **Phylum** | Firmicutes | Firmicutes | Firmicutes | | Firmicutes | Bacteroidetes | | | |
|  | **Class** | Clostridia | Clostridia | Clostridia | | Clostridia | Bacteroidia | | | |
|  | **Order** | Clostridiales | Clostridiales | Clostridiales | | Clostridiales | Bacteroidales | | | |
|  | **Family** | unclassified | Lachnospiraceae | Lachnospiraceae | | Lachnospiraceae | Porphyromonadaceae | | | |
|  | **Genus** | unclassified | unclassified | unclassified | | unclassified | Barnesiella | | | |

## Supplementary Figures


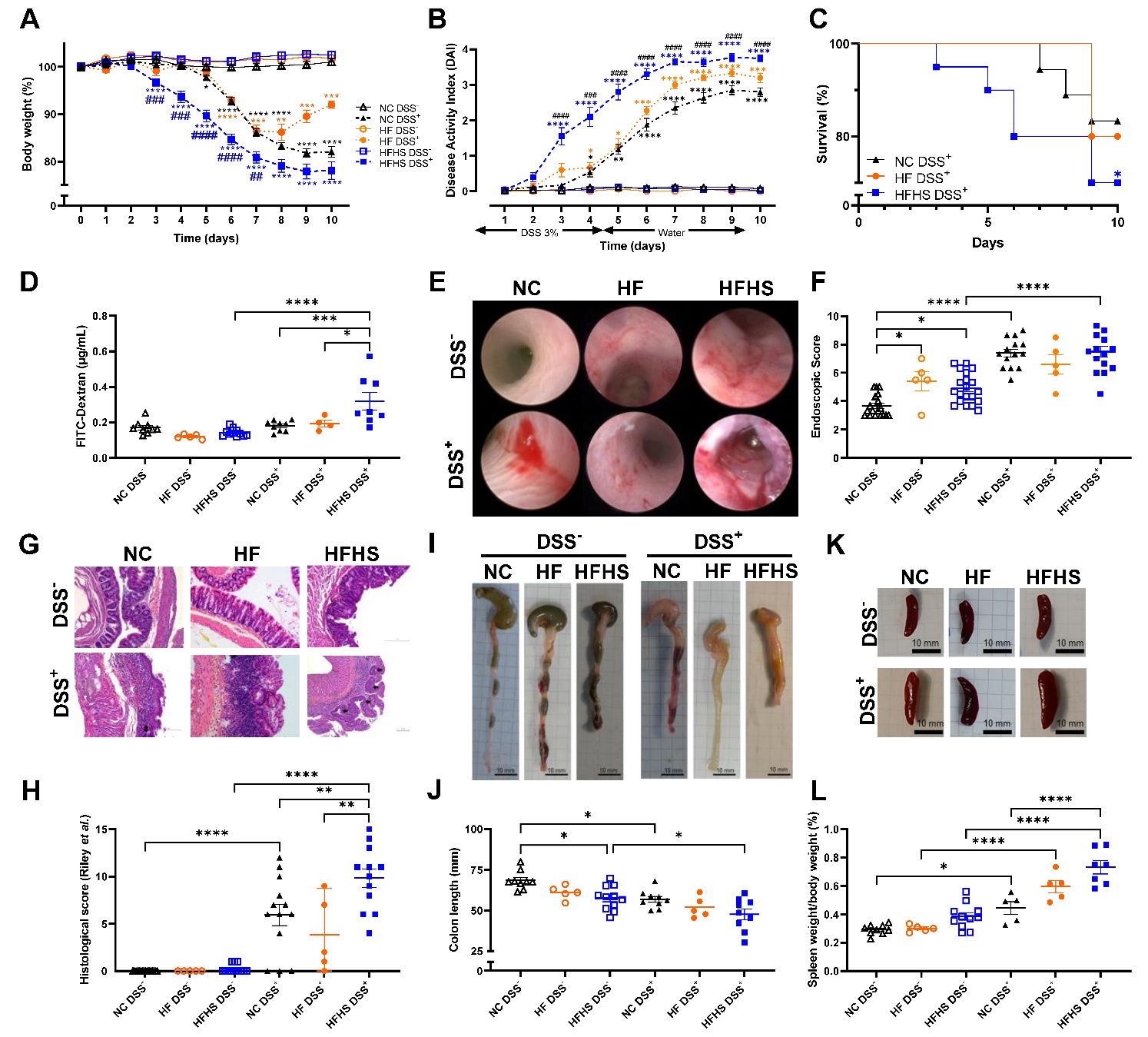


**Figure S1. High-fat high-sucrose diet exacerbates colitis rather than high-fat diet.** Mice were fed 8 weeks normal chow diet (NC) or High Fat (HF) diet or High-Fat High-Sucrose (HFHS) and colitis was induced for half of the animals with dextran sulfate sodium (DSS) in C57BL/6 mice (N ≥ 8 / group). Representative data from two independent experiments is shown. (A) Change in body weight of mice after DSS-induced colitis. (B) Colitis severity was assessed by Disease Activity Index (DAI). (C) Survival rate. (D) Gut permeability assessed by FITC-dextran recovered from the plasma of mice at day 5 of DSS-treatment after oral gavage. (E) Representative colonoscopy images and (F) Ulcerative colitis endoscopic index of severity (UCEIS) on day 10. (G) Representative sections of colon, arrows show epithelial dystrophies. Scale bars, 0.10 mm. (H) Histological scores on day 10. (I) Gross pictures of colons. Scale bars, 10 mm. (J) Colon lengths. (K) Gross pictures of spleens, scale bars: 10 mm. (L) Spleen weight to body weight ratio on day 10 of DSS-treatment. Means +/- SEM are plotted. * *p* < 0.05, ** *p* < 0.01, **** *p* < 0.0001 by ANOVA (two-way for A and B) for parametric data or Kruskal-Wallis if not. # *p* < 0.05, ## p < 0.01, ### *p* < 0. 001 between HFHS and NC groups. For survival rate, Log-rank (Mantel-Cox) test was used, * *p* < 0.05.

**Figure S2 (Separate file). Disturbance of immune response to colitis under high-fat high-sucrose diet.** (A) Immune cell population distribution. Rate of indicated immune cells in the mesenteric lymph nodes and the spleen of mice fed with normal chow diet (NC) or high-fat (HF) or high-fat high-sucrose diet (HFHS) or N = 5 per group. (B-D) Plasma and (E-G) colonic tissue levels of indicated cytokines on day 10 for untreated mice fed with normal chow diet (NC DSS^-^, N=4) or high-fat high-sucrose diet (HFHS DSS^-^, N=6) and colitic mice NC-fed (NC DSS^+^, N=4) or HFHS-fed (HFHS DSS^+^, N≥3) mice. Means +/- SEM are plotted. * *p* < 0.05, ** *p* < 0.01, *** *p* < 0.001, **** *p* < 0.0001 by ANOVA for parametric data or Kruskal-Wallis if not.


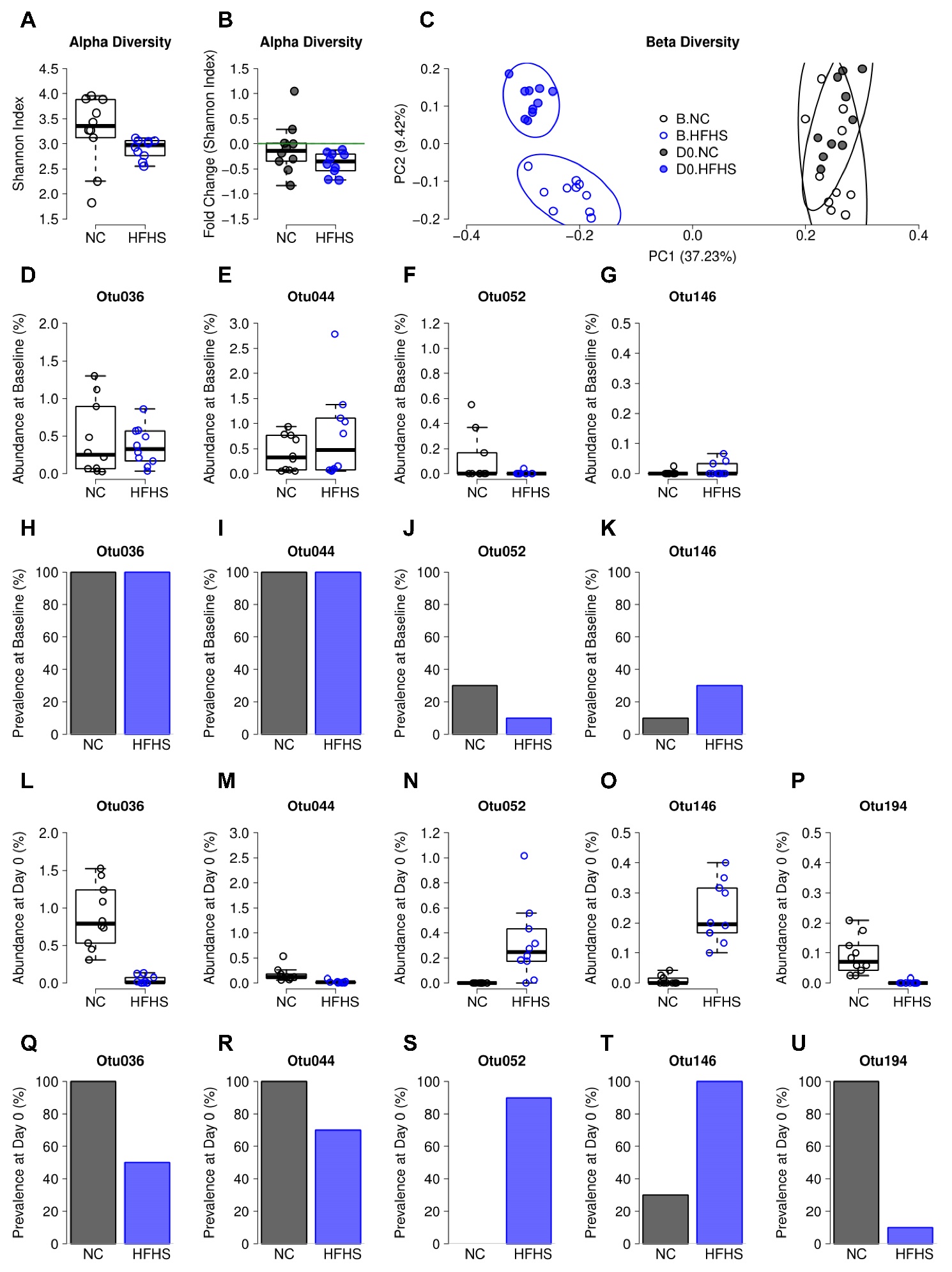


**Figure S3.** **High-fat high-sucrose diet dysbiosis.** (A) α-diversity measured by Shannon index of mice fed with normal chow diet (NC, N=10) *versus* high-fat high-sucrose diet (HFHS, N=9) at baseline (B). (B) Evolution of species richness measured by a fold change of Shannon index at D0 (day zero) relative to B. (C) Principal coordinate analysis (PCoA) of Jaccard β-diversity index showing NC-fed vs HFHS-fed mice, at B and at D0. (D-G) Indicator OTU's abundances at B and (L-P) at D0. (H-K) Corresponding prevalence at B and (Q-U) at D0.


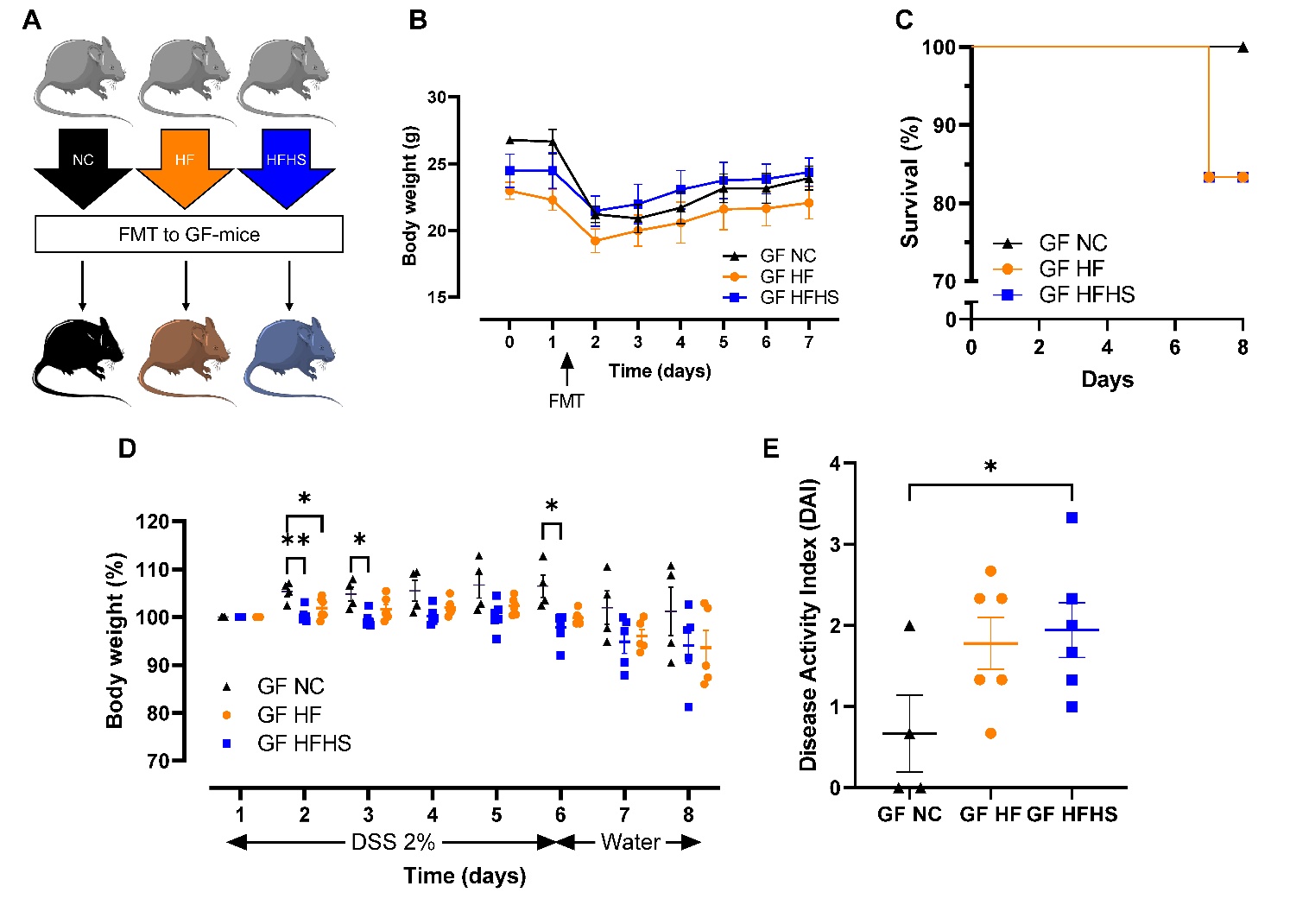


**Figure S4. Colitis worsening of high-fat high-sucrose diet-fed mice is transmissible.** (A) Experimental design. Feces collected from mice fed with high-fat high-sucrose Diet (HFHS) or high-fat (HF) or Normal Chow (NC) during 8 weeks were orally inoculated into Germ-Free (GF)-mice. Seven days after fecal transplant, recipient mice (N = 4 to 6 per group) were treated with 2% DSS for 5 days. (B) Body weight change after Fecal Microbiota Transplantation (FMT) experiment. (C) Survival rate. (D) Body weight change during colitis. (E) Disease Activity Index (DAI) at day 8. Means +/- SEM are plotted. * *p* < 0.05, ** *p* < 0.01, by ANOVA for parametric data or Kruskal-Wallis if not. For survival rate, Log-rank (Mantel-Cox) test was used.


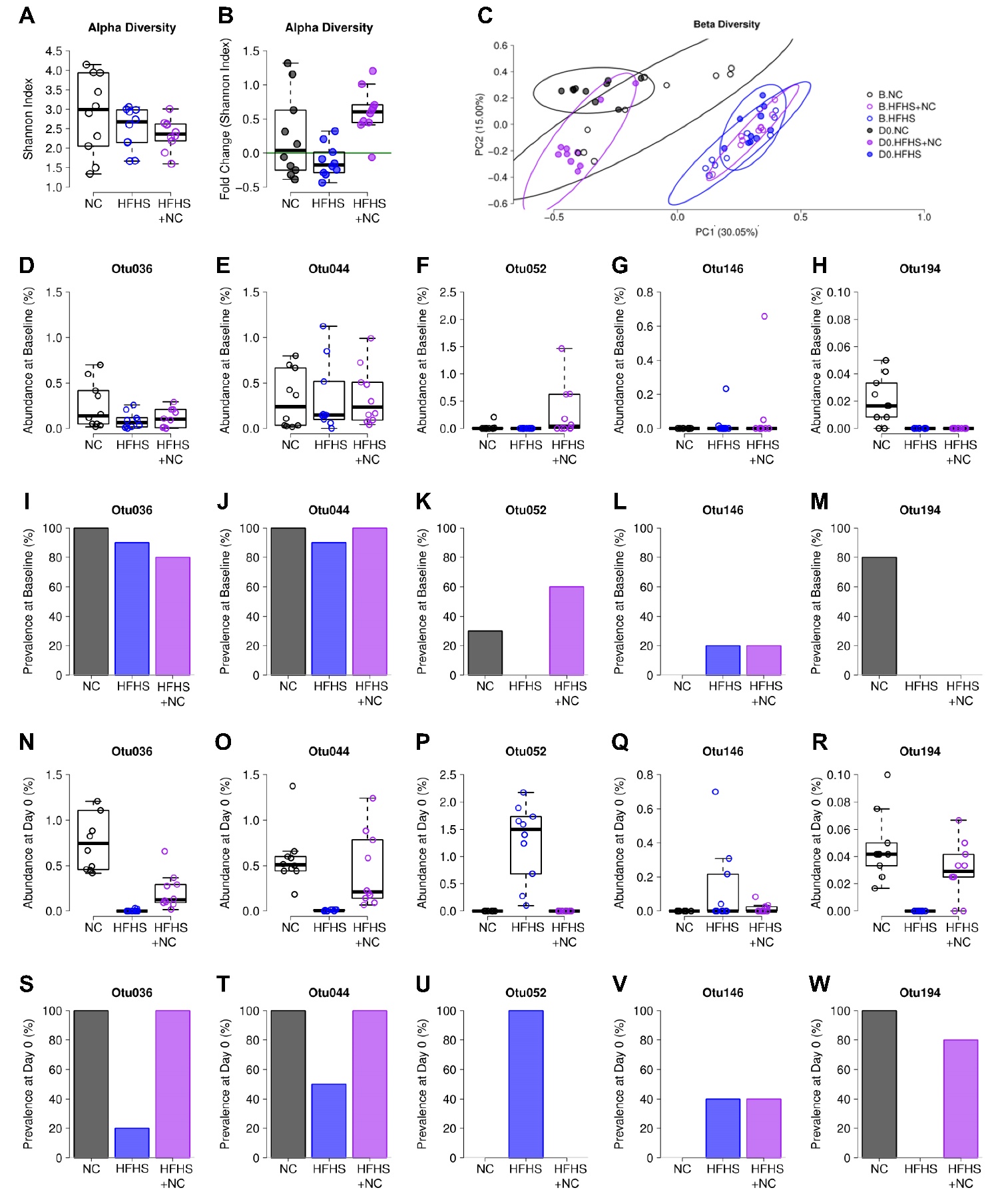


**Figure S5. Normal chow diet reverts some of the high-fat high-sucrose-associated dysbiosis.** (A) α-diversity measured by Shannon index of mice fed with normal chow diet (NC, N=10), high-fat high-sucrose diet (HFHS, N=10) or HFHS+NC (N=10) at baseline (B). (B) Evolution of species richness measured by a fold change of Shannon index at day zero (D0) relative to B. (C) Principal coordinate analysis (PCoA) of Bray-Curtis β-diversity index showing NC-fed, HFHS-fed, or HFHS+NC-fed mice, at B and at D0. (D-H) Abundances at B and n-r at D0, and (I-M) corresponding prevalence at B and (S-W) D0 of the five OTUs identified as indicator for diet in the 8-weeks experiment.


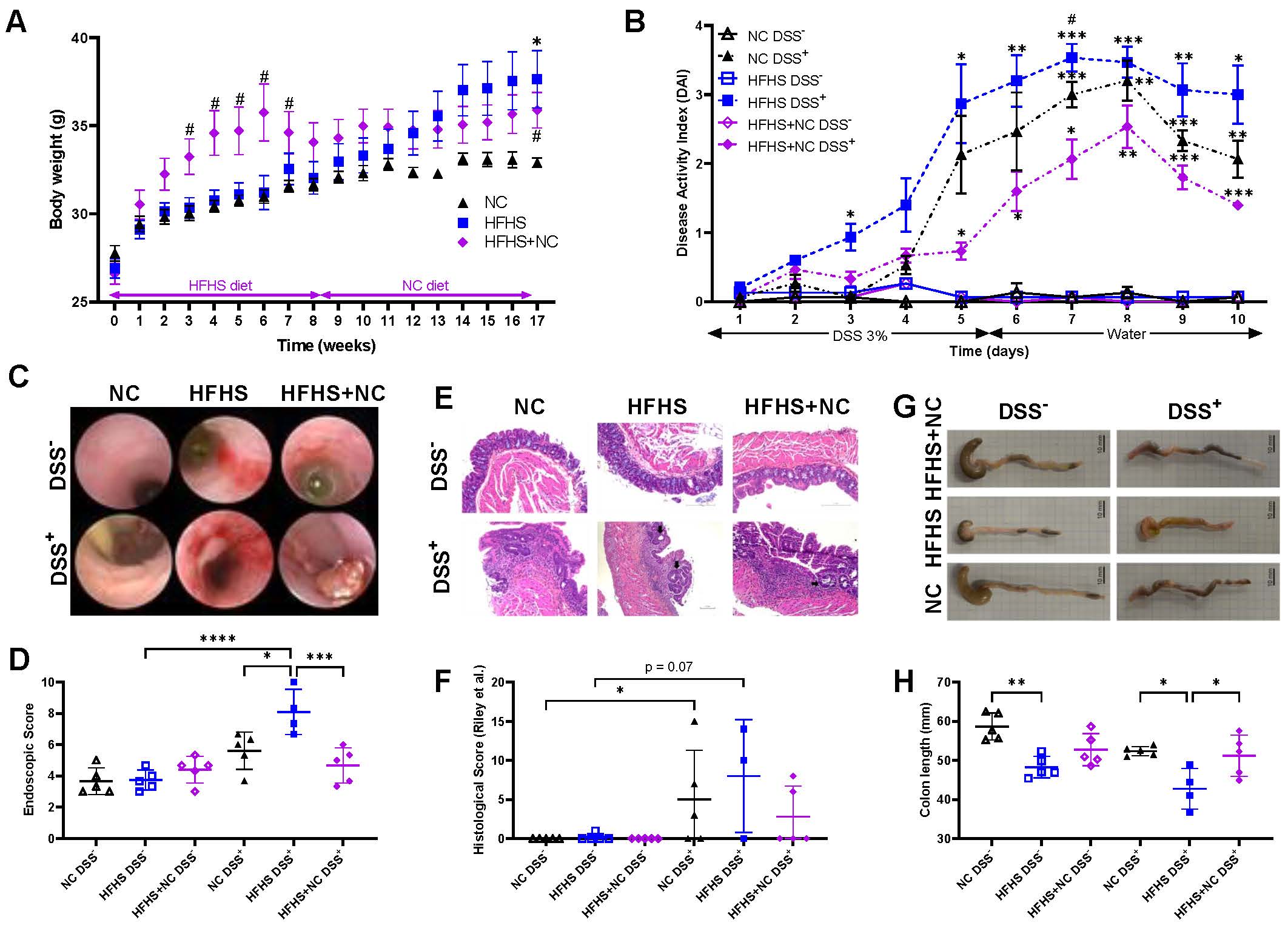


**Figure S6. Cessation of fat and sugar overconsumption restoring during colitis.** Mice were fed 16 weeks normal chow diet (NC) or High-Fat High-Sucrose (HFHS) or 8 weeks HFHS before switching back to NC for 8 weeks. Colitis was induced for half of the animals with dextran sulfate sodium (DSS) in C57BL/6 mice (N ≥ 8 / group). (A) Change in body weight during diet experiment. (B) Colitis severity was assessed by Disease Activity Index (DAI). (C) Representative colonoscopy images and (D) Ulcerative colitis endoscopic index of severity (UCEIS) on day 10. (E) Representative sections of colon, arrows show epithelial dystrophies. Scale bars, 0.10 mm. (F) Histological scores on day 10. (G) Gross pictures of colons. Scale bars, 10 mm. (H) Colon lengths. Means +/- SEM are plotted. * p < 0.05, ** p < 0.01, **** p < 0.0001 by ANOVA for parametric data or Kruskal-Wallis if not. # p < 0.05, ## p < 0.01, ### p < 0. 001 between HFHS and NC groups.

**Figure S7 (Separate file). Microbiota composition of each group.** (A) High-fat high-sucrose Diet mice and (B) HFHS then switching to normal chow diet mice. Biomarker counts (a) Venn-diagram of the OTUs identified as indicator of diet x treatment condition at baseline (B), day zero (D0), and fold change (FC). For D0, the number in brackets refers to OTUs associated with diet x treatment with a *p*.value ≤ 0.10. The dotted black lines indicate the number of indicator OTUs with a *p*.value ≤ 0.05 for one time point and ≤ 0.10 for the other time point. Comparison of the microbial composition of the entire community ("all") and subsets of the community composed only of the indicator OTUs.

**Figure S8 (Separate file). Spleen and mesenteric lymph nodes flow cytometry analysis.** Gating Strategy for flow cytometry analysis in spleen and mesenteric lymph nodes

**Dataset S1 (separate file). Differentially expressed genes between all groups.**

**Dataset S2 (separate file). Scripts for the processing of the raw 16S sequences in usearch and mothur.**

**Dataset S3 (separate file). Number of sequences per sample throughout the processing steps.**

**Dataset S4 (separate file). Formatting scripts for OTU and count Tables.**

**Dataset S5 (separate file). Final OTU and MetaData Tables used for the analysis, together with corresponding script**
