# Supplementary figures and images for: Long-Term Overconsumption of Fat and Sugar Causes a Partially Reversible Pre-inflammatory Bowel Disease State
Source: Front Nutr. 2021 Nov 18;8:758518. doi: 10.3389/fnut.2021.758518 (PMC8637418; doi:10.3389/fnut.2021.758518)

**A**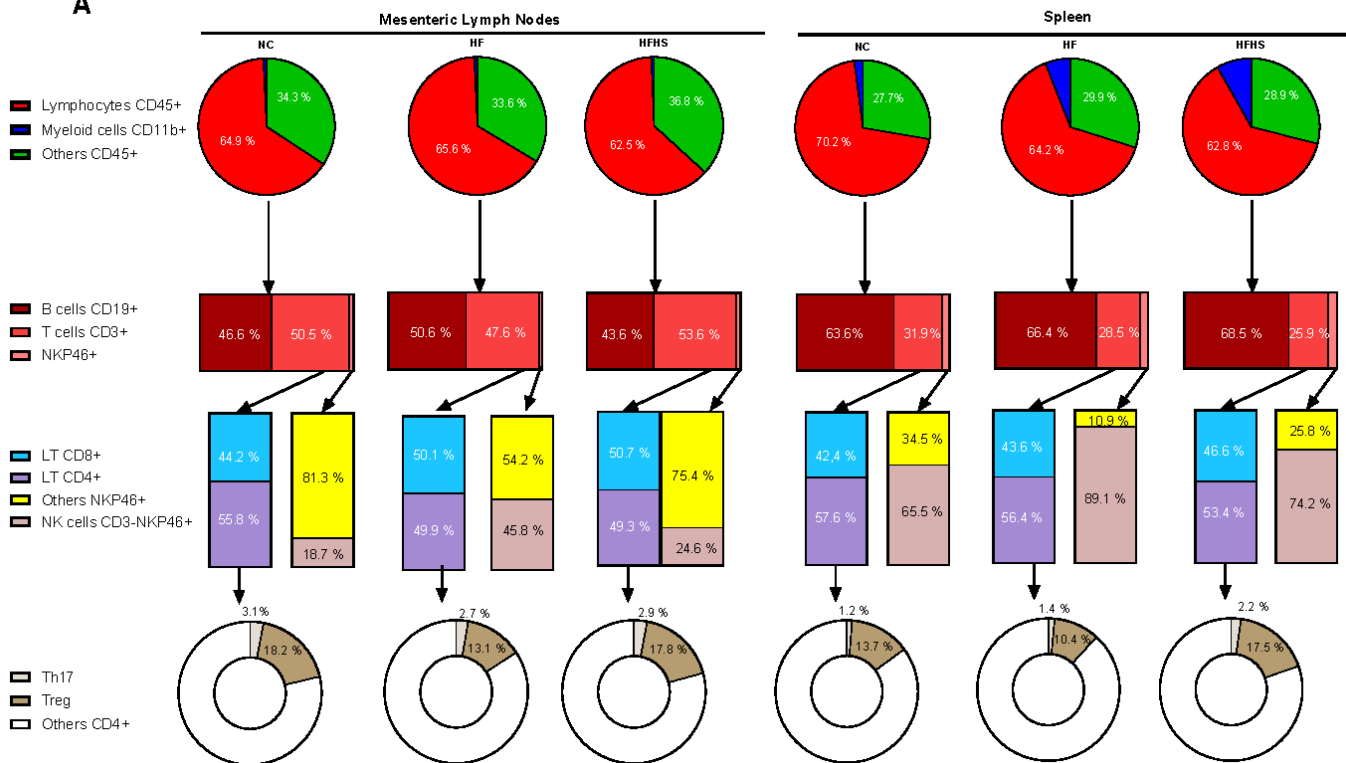**B**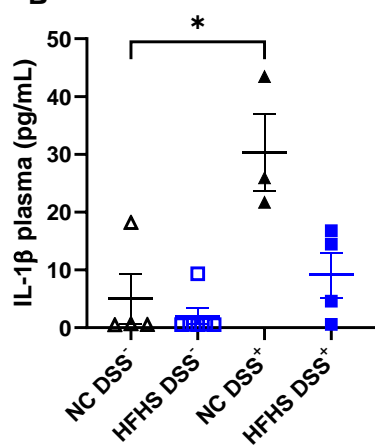**C**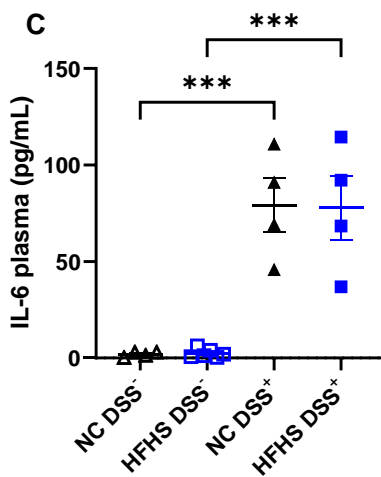**D**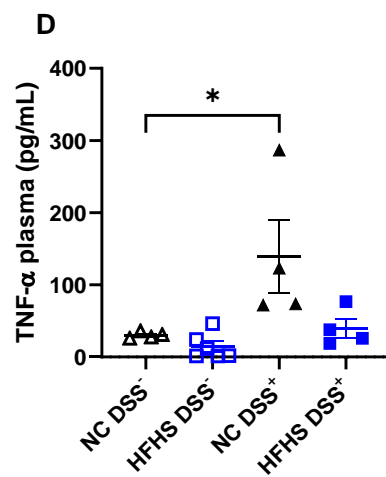**E**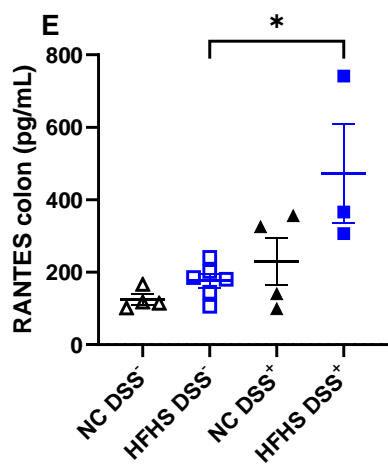**F**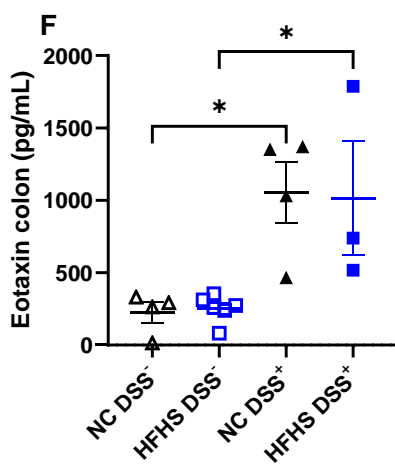**G**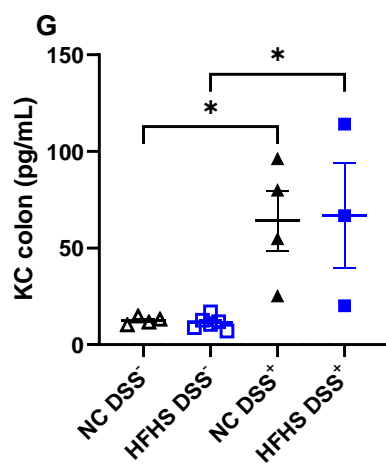

Supplement: Supplementary file 3 [file Image_2.PDF]

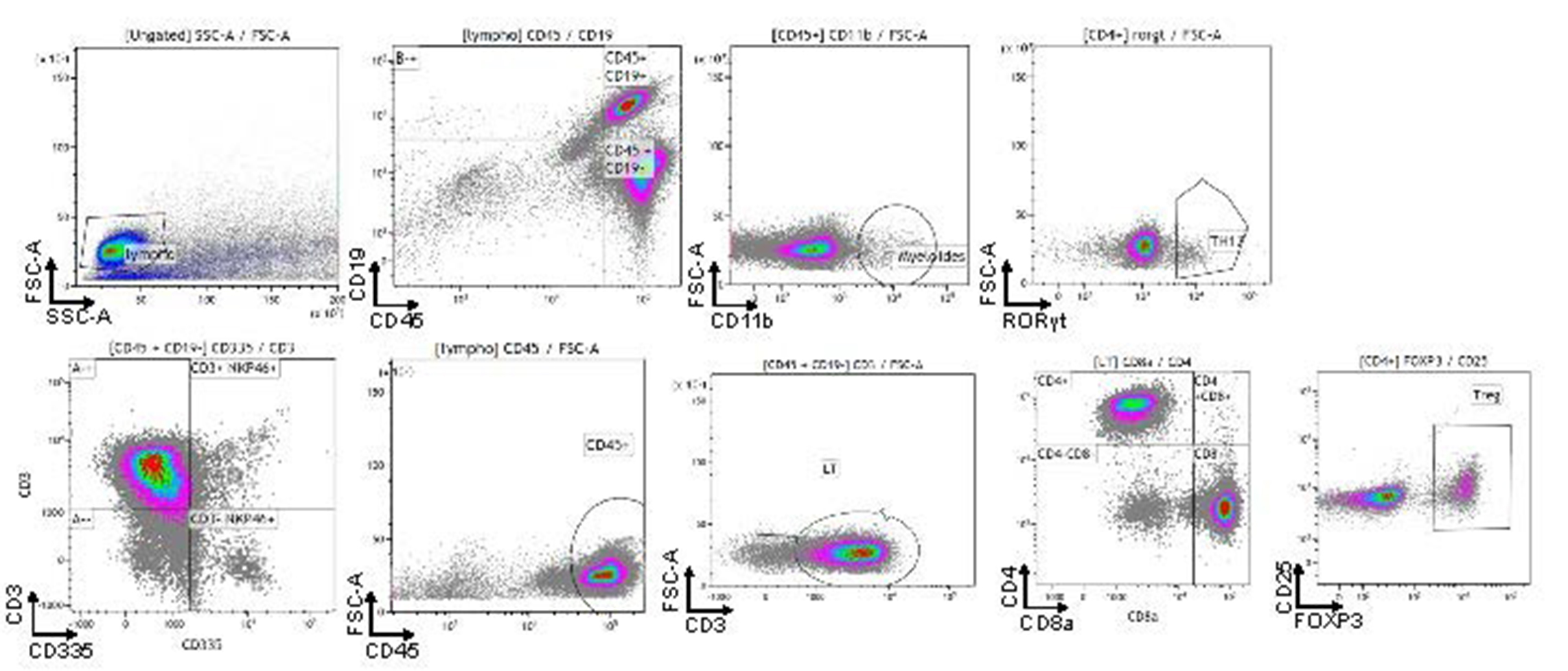

Supplement: Supplementary file 5 [file Image_8.JPEG]
